# AIF-regulated oxidative phosphorylation supports lung cancer development

**DOI:** 10.1038/s41422-019-0181-4

**Published:** 2019-05-27

**Authors:** Shuan Rao, Laura Mondragón, Blanka Pranjic, Toshikatsu Hanada, Gautier Stoll, Thomas Köcher, Peng Zhang, Alexander Jais, Alexander Lercher, Andreas Bergthaler, Daniel Schramek, Katharina Haigh, Valentina Sica, Marion Leduc, Nazanine Modjtahedi, Tsung-Pin Pai, Masahiro Onji, Iris Uribesalgo, Reiko Hanada, Ivona Kozieradzki, Rubina Koglgruber, Shane J. Cronin, Zhigang She, Franz Quehenberger, Helmut Popper, Lukas Kenner, Jody J. Haigh, Oliver Kepp, Malgorzata Rak, Kaican Cai, Guido Kroemer, Josef M. Penninger

**Affiliations:** 1 Department of Thoracic Surgery, Nanfang Hospital, Southern Medical University, Guangzhou, Guangdong China; 2 IMBA, Institute of Molecular Biotechnology of the Austrian Academy of Sciences, 1030 Vienna, Austria; 3 Equipe 11 labellisée Ligue contre le Cancer, Centre de Recherche des Cordeliers, 75006 Paris, France; 4 INSERM, U1138, 75006 Paris, France; 5 Université Paris Descartes, Sorbonne Paris Cité, Paris, France; 6 Metabolomics and Cell Biology Platforms, Gustave Roussy Cancer Campus, 94805 Villejuif, France; 7 Université Sorbonne, 75006 Paris, France; 8 Vienna Biocenter Core Facilities, 1030 Vienna, Austria; 9 Medical Science Research Center, Zhongnan Hospital of Wuhan University, Wuhan, Hubei China; 10 Department of Neuronal Control of Metabolism, Max Planck Institute for Metabolism Research, Cologne, Germany; 11 Research Center for Molecular Medicine of the Austrian Academy of Sciences, Vienna, Austria; 12 Lunenfeld-Tanenbaum Research Institute, Mount Sinai Hospital, 600 University Avenue, Toronto, Canada; 13 Vascular Cell Biology Unit, Department for Molecular Biomedical Research, VIB, Ghent, Belgium; 14 Department of Biomedical Molecular Biology, Ghent University, Ghent, Belgium; 15 Department of Pharmacology and Therapeutics, Rady Faculty of Health Sciences, University of Manitoba, Winnipeg, Canada; 16 Gustave Roussy Cancer Campus, Villejuif, France; 17 Faculty of Medicine, Université Paris-Saclay, Kremlin-Bicêtre, France; 18 INSERM, U1030 Villejuif, France; 19 Department of Cardiology, Renmin Hospital of Wuhan University, Wuhan, Hubei China; 20 Institute for Medical Informatics, Statistics and Documentation, Medical University Graz, Graz, Austria; 21 Center for Diagnostics and Research in Molecular Biomedicine, Pathology Institute for Diagnostics and Research, Medical University Graz, Graz, Austria; 22 Department of Experimental Pathology and Pathology of Laboratory Animals, Medical University Vienna and University of Veterinary Medicine Vienna, Vienna, Austria; 23 Ludwig Boltzmann Institute for Cancer Research (LBI-CR), Vienna, Austria; 24 INSERM, UMR1141, Hopital Robert Debre 48 Boulevard Serurier, 75019 Paris, France; 25 Pôle de Biologie, Hôpital Européen Georges Pompidou, AP-HP, Paris, France; 26 Suzhou Institute for Systems Biology, Chinese Academy of Sciences, Suzhou, Jiangsu China; 27 Department of Women’s and Children’s Health, Karolinska Institute, Karolinska University Hospital, Stockholm, Sweden; 28 Department of Medical Genetics, Life Sciences Institute, University of British Columbia, Vancouver, Canada

**Keywords:** Non-small-cell lung cancer, Mechanisms of disease

## Abstract

Cancer is a major and still increasing cause of death in humans. Most cancer cells have a fundamentally different metabolic profile from that of normal tissue. This shift away from mitochondrial ATP synthesis via oxidative phosphorylation towards a high rate of glycolysis, termed Warburg effect, has long been recognized as a paradigmatic hallmark of cancer, supporting the increased biosynthetic demands of tumor cells. Here we show that deletion of apoptosis-inducing factor (AIF) in a *Kras*^*G12D*^-driven mouse lung cancer model resulted in a marked survival advantage, with delayed tumor onset and decreased malignant progression. Mechanistically, *Aif* deletion leads to oxidative phosphorylation (OXPHOS) deficiency and a switch in cellular metabolism towards glycolysis in non-transformed pneumocytes and at early stages of tumor development. Paradoxically, although *Aif*-deficient cells exhibited a metabolic Warburg profile, this bioenergetic change resulted in a growth disadvantage of *Kras*^*G12D*^-driven as well as *Kras* wild-type lung cancer cells. Cell-autonomous re-expression of both wild-type and mutant AIF (displaying an intact mitochondrial, but abrogated apoptotic function) in *Aif*-knockout *Kras*^*G12D*^ mice restored OXPHOS and reduced animal survival to the same level as AIF wild-type mice. In patients with non-small cell lung cancer, high AIF expression was associated with poor prognosis. These data show that AIF-regulated mitochondrial respiration and OXPHOS drive the progression of lung cancer.

## Introduction

AIF (apoptosis-inducing factor) was first cloned as a caspase-independent death effector released from mitochondria.^1^ Since then, multiple publications pointed to an important, albeit not essential, function of AIF in several cell death scenarios in multiple species including mouse, *S. cerevisiae*, *C. elegans*, and *D. melanogaster*.^2–4^ Importantly, similar to the key cell death effector molecule cytochrome c, AIF not only contributes to cell death pathways, but also exerts a vital housekeeping function inside mitochondria, where it determines the rate of oxidative phosphorylation (OXPHOS) via posttranscriptional regulation of complex I proteins in the mitochondrial respiratory chain.^3,5–8^ Mice with tissue-specific *Aif* deletions or a hypomorph *Aif* mutation exhibit organ-specific complex I deficiency and enhanced glycolysis, confirming a key function for AIF in mitochondrial respiration.^3,5–8^ Since AIF is a protein with a dual function in cell death and OXPHOS, we tested whether genetic modulation of *Aif* would affect lung cancer tumorigenesis.

Cancer cells have a fundamentally different metabolic profile from that of normal tissue and this shift away from mitochondrial ATP synthesis via OXPHOS towards a high rate of glycolysis has long been recognized as a hallmark of cancer cells.^9,10^ This glycolytic switch was termed the “Warburg effect”, named after Otto Warburg who discovered this phenomenon in 1923 and later suggested it to constitute a fundamental cause of cancer.^11^ The Warburg effect has been proposed to support proliferation and the increased biosynthetic demands of cancer cells.

In this study, we use a genetic murine system to directly decrease the function of the respiratory chain (and hence to inhibit OXPHOS) in *Kras*^*G12D*^-induced lung carcinogenesis by knocking out AIF. According to the Warburg hypothesis, this manipulation should accelerate oncogenesis. We indeed observed a bioenergetic Warburg shift in primary, non-transformed AIF mutant pneumocytes as well as at early stages of tumorigenesis. However, we observed that ablation of AIF reduced *Kras*^*G12D*^-driven lung carcinogenesis. Most importantly, cell-autonomous, engineered re-introduction of wild-type (WT) AIF or a mitochondria-confined mutant of AIF (that loses its extramitochondrial pro-death function) was sufficient to re-establish OXPHOS and *Kras*^*G12D*^-driven carcinogenesis.

To expand our findings, we analyzed both *Kras* mutant and WT human lung cancer cell lines from non-small cell lung cancer (NSCLC) patients. Interestingly, depletion of AIF resulted in impaired growth and clonogenic potential of all these human lung cancer cells, thereby confirming our conclusions with the genetic murine lung cancer data. Furthermore, by including lung cancer patients’ data, we have demonstrated that both AIF mRNA and protein expression correlate with survival, and high levels of AIF are associated with poor prognosis. Surprisingly, by analyzing different NSCLC cohorts, we found that most genes encoding the complex I subunits of mitochondrial respiratory chain or their assembly factors were overexpressed in NSCLC tissues as compared to normal adjacent lung tissues. All these findings collectively confirm and strengthen the conclusion that OXPHOS is supportive for lung cancer development in general, regardless of genetic background.

## Results

### Reduced lung cancer in *Aif*-deficient *Kras*^*G12D*^ mice

**Fig. 1:**
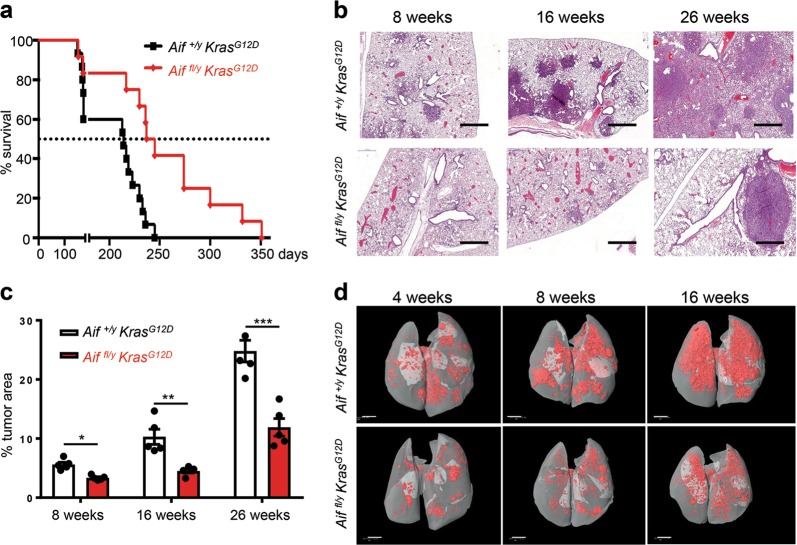
Reduced lung cancer in *Aif*-deficient *Kras*^*G12D*^ mice. **a** *Aif* deletion significantly prolongs the survival of mice infected with Ad5-CMV-Cre in comparison to their *Aif* WT controls. Kaplan–Meier plot. *P* = 0.0022 (log rank test) for *Aif*^*fl/y*^ *Kras*^*G12D*^ (*n* = 12) versus *Aif*^*+/y*^ *Kras*^*G12D*^ (*n* = 15) littermates. **b** Representative lung tumor sections (H&E staining) in *Aif*^*fl/y*^ *Kras*^*G12D*^ and *Aif*^*+/y*^ *Kras*^*G12D*^ littermates at the indicated time points after Ad5-CMV-Cre inhalation. Scale bar, 2 mm. **c** Quantification of overall tumor burden. Total tumor areas comprising hyperplasia, adenomas, and adenocarcinomas, were scored automatically by a Definiens software algorithm. Three planes from each lung were stained with H&E and analyzed in a blinded fashion. Data are shown as means ± SEM. *P < 0.05; **P < 0.01; ***P < 0.001 (Student’s *t*-test). *n* = 5 per genotype for each time point. **d** Micro-CT analysis of lung tumors of *Aif*^*fl/y*^ *Kras*^*G12D*^ and *Aif*^*+/y*^ *Kras*^*G12D*^ littermate mice, analyzed at the indicated weeks after Ad5-CMV-Cre inhalation. Representative data from individual mice are shown. Scale bar, 2 mm

To determine the role of AIF in lung cancer, we crossed *Aif* ^*fl/fl*^ mice^3^ with the *Lox-Stop-Lox-Kras*^*G12D*^ strain. *Lox-Stop-Lox-Kras*^*G12D*^ mice develop non-small-cell lung carcinomas (NSCLCs) upon Cre deletion and induction of the mutant *Kras*^*G12D*^ allele in a stepwise process that leads from epithelial hyperplasia to benign adenomas and malignant adenocarcinomas.^12,13^ We achieved expression of *Kras*^*G12D*^ and simultaneous deletion of *Aif* following adenoviral delivery of Cre recombinase (AdCre) through inhalation using the Ad5-CMV-Cre or Ad5-mSPC-Cre virus (Supplementary information, Fig. S1a). The *Aif* gene is located on the X chromosome. Thus, male mice expressing oncogenic *Kras*^*G12D*^ born from heterozygous *Aif*^ *+* */fl*^ mothers develop tumors that are either knockout for *Aif* (*Aif*^*fl/y*^*Kras*^*G12D*^), or normally express AIF (*Aif*^*+/y*^ *Kras*^*G12D*^) as littermate controls_._ Intriguingly, loss of AIF in the *Kras*^*G12D*^-driven lung cancer model resulted in significantly prolonged survival as compared to control *Aif*^*+/y*^ *Kras*^*G12D*^ littermates (Fig. 1a). Quantification of overall tumor burden revealed a significant decrease of the tumor areas in the lungs of *Aif*^*fl/y*^ *Kras*^*G12D*^ mice compared to *Aif*^*+/y*^ *Kras*^*G12D*^ controls at all time-points analyzed (Fig. 1b, c). *Aif*-deficient lung cancers showed efficient deletion of *Aif*, as determined by qPCR of micro-dissected tumors and by immunohistochemistry to detect loss of AIF protein (Supplementary information, Fig. S1b, c). As expected from previous studies,^13^ the tumors arose from surfactant protein (SP-C) expressing type II alveolar pneumocytes (Supplementary information, Fig. S1d). AIF deletion, which is known to compromise the function of respiratory chain complex I,^5,8^ did not affect the integrity of respiratory chain supercomplexes, as analyzed by native gel electrophoresis (Supplementary information, Fig. S1e). These results show that genetic inactivation of *Aif* results in markedly reduced *Kras*^*G12D*^-driven lung tumorigenesis.

We next assessed tumor initiation and staged the malignant progression of the lung cancers. Four weeks after Ad5-CMV-Cre inhalation, micro-CT already revealed markedly reduced tumor foci in *Aif*^*fl/y*^ *Kras*^*G12D*^ mice, and this reduction of tumor foci was observed throughout the entire observation period (Fig. 1d). Using established histopathological criteria^14^ at 4 weeks after Ad5-CMV-Cre inhalation, *Aif*^*+/y*^ *Kras*^*G12D*^ control mice harbored multiple hyperplastic lesions and even small solid adenomas, whereas at this time point only small hyperplastic regions were observed in *Aif*^*fl/y*^ *Kras*^*G12D*^ mice (Supplementary information, Fig. S2a). Eight weeks after Ad5-CMV-Cre inhalation, we again observed significantly less hyperplastic regions as well as reduced numbers of adenomas in *Aif*^*fl/y*^ *Kras*^*G12D*^ mice as compared to *Aif*^*+/y*^ *Kras*^*G12D*^ littermates (Supplementary information, Fig. S2b). At 16 weeks after Ad5-CMV-Cre infection, inactivation of *Aif* resulted in markedly reduced progression to adenocarcinomas (Supplementary information, Fig. S2c). Due to the large size of lung adenocarcinomas at 26 weeks after Ad5-CMV-Cre inhalation, it was not possible to reliably count individual tumors at this time point. Proliferation, as detected by Ki67 staining, was significantly reduced in hyperplastic regions, as well as in early (4 weeks after Ad5-CMV-Cre inhalation) adenomas from *Aif*^*fl/y*^ *Kras*^*G12D*^ mice (Supplementary information, Fig. S2d–f). Moreover, we observed enhanced expression of the senescence marker promyelocytic leukemia protein (PML) (Supplementary information, Fig. S2g, h), the expression of which correlates inversely with the malignancy and proliferative index of tumors.^15^ We detected only a few apoptotic cells (that stained positively for cleaved caspase-3) in *Kras*^*G12D*^-induced lung tumors from both *Aif*^*fl/y*^ *Kras*^*G12D*^ and *Aif*^*+/y*^ *Kras*^*G12D*^ mice, confirming previous reports on the anti-apoptotic potential of oncogenic *Kras*^*G12D*^.^15^ Importantly, we did not find significant differences in the percentages of apoptotic cells among the different cohorts at all time-points analyzed (data not shown). Thus, loss of AIF delays *Kras*^*G12D*^-driven lung tumor initiation and malignant progression to adenocarcinomas.

### Loss of AIF compromises OXPHOS and impairs mitochondrial structure

**Fig. 2:**
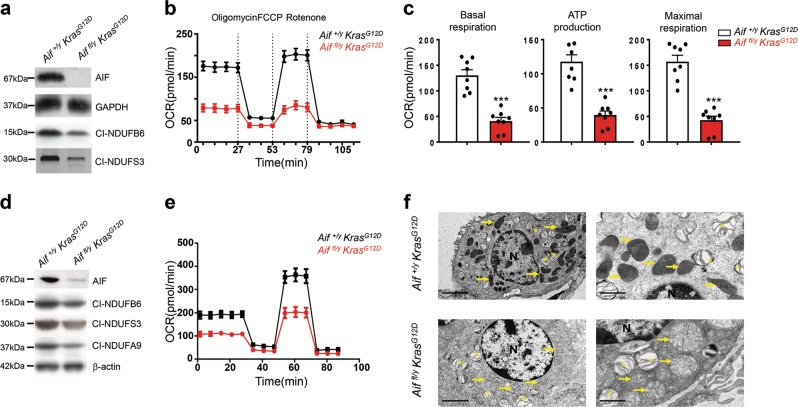
Loss of AIF compromises OXPHOS and impairs mitochondrial structure in lung tumor cells. **a** Western blotting for AIF protein and the indicated OXPHOS complex I proteins in primary pneumocytes isolated from *Aif*^*fl/y*^ *Kras*^*G12D*^ and *Aif*^*+/y*^ *Kras*^*G12D*^ mice and consequently transfected with Ad5-CMV-Cre-eGFP in vitro. GAPDH was used as a loading control. **b**, **c** Representative OCR (**b**) and comparison (means ± SEM) of basal respiration, ATP production and maximal respiration (**c**) in primary pneumocytes isolated from *Aif*^*fl/y*^ *Kras*^*G12D*^ and *Aif*^*+/y*^ *Kras*^*G12D*^ mice and consequently transfected with Ad5-CMV-Cre in vitro_._ **d** Western blotting for AIF protein and the indicated OXPHOS complex I proteins in pneumocytes isolated from *Aif*^*fl/y*^ *Kras*^*G12D*^ and *Aif*^*+/y*^ *Kras*^*G12D*^ mice 6 weeks after Ad5-CMV-Cre inhalation. β-actin was used as a loading control. **e** Representative OCR analysis of purified transformed pneumocytes isolated from *Aif*^*fl/y*^ *Kras*^*G12D*^ and *Aif*^*+/y*^ *Kras*^*G12D*^ mice 6 weeks after Ad5-CMV-Cre inhalation. **f** Representative electron microscopy images for tumor tissues isolated from *Aif*^*+/y*^ *Kras*^*G12D*^ (upper panels) and *Aif*^*fl/y*^ *Kras*^*G12D*^ mice (lower panels) 18 weeks after Ad5-CMV-Cre inhalation. Note normal mitochondrial morphology with mostly intact cristae in AIF-competent tumors in contrast to swollen mitochondria with notable cristolysis in *Aif*-deficient lung tumor tissues (yellow arrows). Asterisks indicate lamellar bodies (*Corpuscula lamellariae*), rare cell organelles containing surfactant lipoproteins characteristic for type II pneumocytes. N indicates nuclei. Scale bars, 5 μm for left panel and 2 μm for right panel

As AIF affects the onset and early stages in tumor development, we isolated primary pneumocytes from *Aif*^*fl/y*^ *Kras*^*G12D*^ and *Aif*^*+/y*^ *Kras*^*G12D*^ littermates and subsequently infected the cultured cells with Ad5-CMV-Cre-eGFP to activate *Kras*^*G12D*^ expression and concomitantly delete *Aif* (Fig. 2a; Supplementary information, Fig. S3a). Deletion of AIF, as expected from previous studies,^6,8^ also resulted in a consequent reduction of proteins in the mitochondrial complex I (Fig. 2a). *Aif* mutant primary pneumocytes were then analyzed for their oxygen consumption rate (OCR)^16^ to assess mitochondrial respiration. *Aif*^*fl/y*^ *Kras*^*G12D*^ cells consumed oxygen at a significantly lower basal level, produced much less ATP as well as exhibited a limited OCR increase in response to the uncoupler carbonylcyanide-p trifluoromethoxyphenylhydrazone (FCCP), yielding a reduced maximal respiratory capacity (as an indicator of the level of OXPHOS) (Fig. 2b, c). In addition, *Aif* deficiency caused a decrease in mitochondrial spare respiration capacity with a simultaneous reduction of proton leakage (Supplementary information, Fig. S3b).

We next isolated primary transformed pneumocytes from *Aif*^*fl/y*^ *Kras*^*G12D*^ and *Aif*^*+/y*^ *Kras*^*G12D*^ littermates 6 weeks after in vivo Ad5-CMV-Cre infection. Of note, the mice were infected with 5 times higher viral titer than the normal concentration (2.5 × 10^7^ PFU) to ensure that the majority of pneumocytes were infected (Fig. 2d). Similar to our short-term cultures, in vivo Ad5-CMV-Cre treatment resulted in reduced basal respiration, ATP production, maximal respiration, spare respiratory capacity as well as proton leakage (Fig. 2e; Supplementary information, Fig. S3c). We also characterized mitochondria in freshly dissected tumor tissue from *Aif*^*fl/y*^ *Kras*^*G12D*^ and *Aif*^*+/y*^ *Kras*^*G12D*^ mice 16 weeks after Ad5-CMV-Cre inhalation. No difference was found in mitochondria numbers between AIF-competent and -deficient tumor cells; however, *Aif*^*fl/y*^ *Kras*^*G12D*^ tumor cells exhibited swollen mitochondria with dramatically decreased and disordered cristae structures (Fig. 2f). Thus, loss of AIF results in not only a general impairment of OXPHOS, but also an abnormal mitochondrial morphology.

### Aif deficiency enhances glycolysis and sensitivity to glucose deprivation

**Fig. 3:**
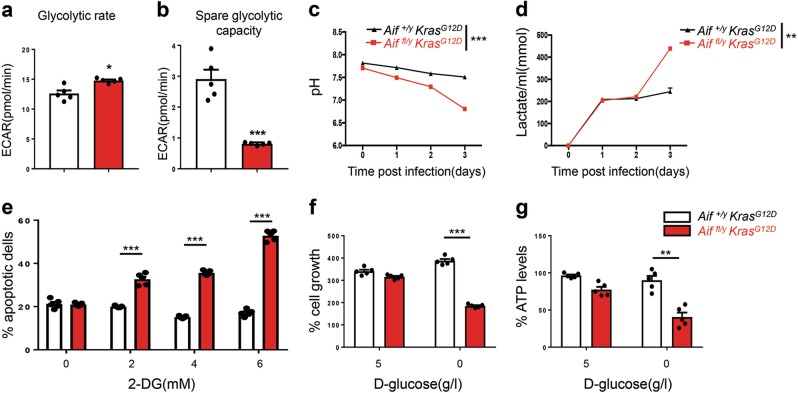
AIF deficiency enhances glycolysis and sensitivity to glucose deprivation. **a**, **b** ECAR in pneumocytes isolated from *Aif*^*+/y*^ *Kras*^*G12D*^ and *Aif*^*fl/y*^ *Kras*^*G12D*^ mice 6 weeks after Ad5-CMV-Cre inhalation. Basal glycolytic rate after stimulation with glucose (**a**) and change in ECAR over baseline after oligomycin treatment (**b**). *P < 0.05; ***P < 0.001 (Unpaired two-sided *t*-test). **c**, **d** pH measurements (**c**) and lactate production (**d**) in the culture media of primary pneumocytes isolated from *Aif*^*+/y*^ *Kras*^*G12D*^ and *Aif*^*fl/y*^ *Kras*^*G12D*^ mice 6 weeks after Ad5-CMV-Cre inhalation. Cells were seeded on day 0 with a density of 2.5 × 10^5^ cells/well in a 6-well plate. Data are shown as means ± SEM. *n* = 5 per genotype. **P < 0.01; ***P < 0.001 (Two-way ANOVA test). **e** *Aif*^*+/y*^ *Kras*^*G12D*^ and *Aif*^*fl/y*^ *Kras*^*G12D*^ pneumocytes were cultured for 72 h in the presence of the indicated concentrations of 2-DG followed by staining with PI to determine the frequency of dead cells. ***P < 0.001 (two-way ANOVA, Bonferroni’s post hoc test). **f** *Aif*^*+/y*^ *Kras*^*G12D*^ and *Aif*^*fl/y*^ *Kras*^*G12D*^ pneumocyte growth in the absence of glucose. Cells were cultured for three days in the presence (5 g/L) or absence of glucose and their viability was determined. **g** Reduced ATP production in *Aif*^*fl/y*^ *Kras*^*G12D*^ cells upon glucose withdrawal. Pneumocytes were cultured for 36 h in the absence or presence of glucose and intracellular ATP levels were determined among the viable cell fractions. ATP content was normalized to the protein concentration of the samples. Data are shown as means±SEM. *n* = 5 per genotype. *P < 0.05; **P < 0.01; ***P < 0.001 (two-way ANOVA, Bonferroni’s post hoc test)

Upon stimulation of glycolysis by addition of glucose, the basal extracellular acidification rate (ECAR, a surrogate of lactate release), was higher in *Aif*^*fl/y*^ *Kras*^*G12D*^ pneumocytes as compared to *Aif*^*+/y*^ *Kras*^*G12D*^ control cells (Fig. 3a). After addition of oligomycin, which suppresses ATP production by OXPHOS, the spare glycolytic activity increased in both *Aif*^*fl/y*^ *Kras*^*G12D*^ and *Aif*^*+/y*^ *Kras*^*G12D*^ cells, albeit significantly more in *Aif*^*+/y*^ *Kras*^*G12D*^ pneumocytes (Fig. 3b), indicating that glycolysis in *Aif*^*fl/y*^ *Kras*^*G12D*^ cells is occurring at a close-to-maximum rate that is barely increased when ATP synthesis from oxidative metabolism is inhibited. Increased glycolysis was also observed in ex vivo Ad5-mSPC-Cre-treated A*if*^*fl/y*^ *Kras*^*G12D*^ pneumocytes, as determined by measuring extracellular pH and the release of lactate into the culture media over several days or by quantifying ECAR in short-term experiments (Fig. 3c, d). An inhibitor of glycolysis, 2-D-deoxyglucose (2-DG), was next utilized to determine whether the survival of A*if*^*fl/y*^ *Kras*^*G12D*^ pneumocytes depends on glycolysis. Control *Aif*^*+/y*^ *Kras*^*G12D*^ cells were not affected by 72-h incubation with 2-DG, even at a concentration of 6 mM, whereas *Aif*^*fl/y*^ *Kras*^*G12D*^ cells exhibited a dose-dependent increase in mortality in response to 2-DG (Fig. 3e). Similarly, glucose withdrawal had no apparent effect on *Aif*^*+/y*^ *Kras*^*G12D*^ cells, yet compromised the growth of *Aif*^*fl/y*^ *Kras*^*G12D*^ cells (Fig. 3f). Of note, upon culturing in the presence of glucose, the cell numbers and rates of cell death (determined by propidium iodide (PI) staining) of both *Aif*^*+/y*^ *Kras*^*G12D*^ and *Aif*^*fl/y*^ *Kras*^*G12D*^ pneumocytes were comparable (data not shown). Finally, we observed a marked decline in intracellular ATP levels in *Aif*^*fl/y*^*Kras*^*G12D*^ pneumocytes cultured in glucose-depleted conditions (Fig. 3g). These results indicate that *Aif* deletion leads to OXPHOS deficiency and a switch in cellular metabolism towards glycolysis. In *Aif*^*fl/y*^ *Kras*^*G12D*^ pneumocytes, OXPHOS marginally contributes to the synthesis of ATP, which is mostly provided by glycolysis, contrasting with *Aif*^*+/y*^ *Kras*^*G12D*^ cells, in which most of the ATP is generated by OXPHOS.

### Stable *Aif* depletion impairs clonogenic potential and proliferation of both *KRAS* WT and *KRAS* mutant human lung cancer cells

**Fig. 4:**
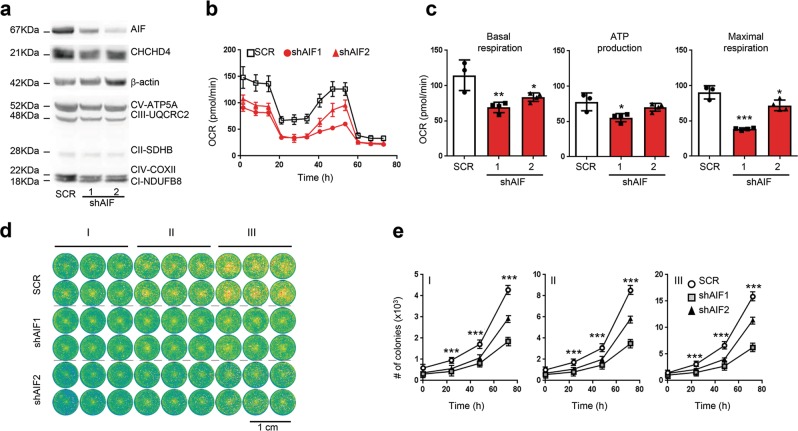
AIF knockdown results in suppression of OXPHOS, clonogenic potential and cell proliferation in human NSCLC A549 lung tumor cells. **a** Cellular extracts from A549 clones, generated by lentiviral transduction with shRNA scramble (SCR) or two different shRNA constructs targeting AIF (shAIF1 and shAIF2), were analyzed by immunoblot for the abundance of the indicated proteins. See Supplementary information, Fig. S6 for quantification. **b** Representative OCR of A549 SCR, shAIF1 and shAIF2 clones under basal conditions or following the addition of 1 μM oligomycin, 1.5 μM of the uncoupler FCCP or 0.5 μM of the electron transport inhibitor rotenone (*n* = 5). **c** Quantification of basal respiration, ATP consumption and maximal respiration levels for SCR, shAIF1, and shAIF2 A549 clones. Results were normalized versus a SCR clone cells/well number and expressed as means ± SEM (experiment was done in triplicate with similar results). **d** Representative cell growth assay of SCR, shAIF1 and shAIF2 A549 lung tumor clones (I, 500 cells/well; II, 1000 cells/well, and III, 2000 cells/well), analyzed by GFP fluorescence at 72 h post-seeding. **e** The indicated SCR, shAIF1 and shAIF2 A549 clones were plated (I, 500 cells/well; II, 1000 cells/well and III, 2000 cells/well) and colony numbers were quantified by GFP fluorescence at 0, 24, 48 and 72 h post-seeding. Values are means ± SEM of a representative experiment containing 24 repeats of each condition (experiment was done in triplicate with similar results). Unpaired two-sided *t*-test, *P < 0.05; **P < 0.01; ***P < 0.001 in case of immunoblot and oxygen consumption studies and two-way ANOVA and Bonferroni’s post hoc test in case of cell proliferation studies, compared to control SCR cells

**Fig. 5:**
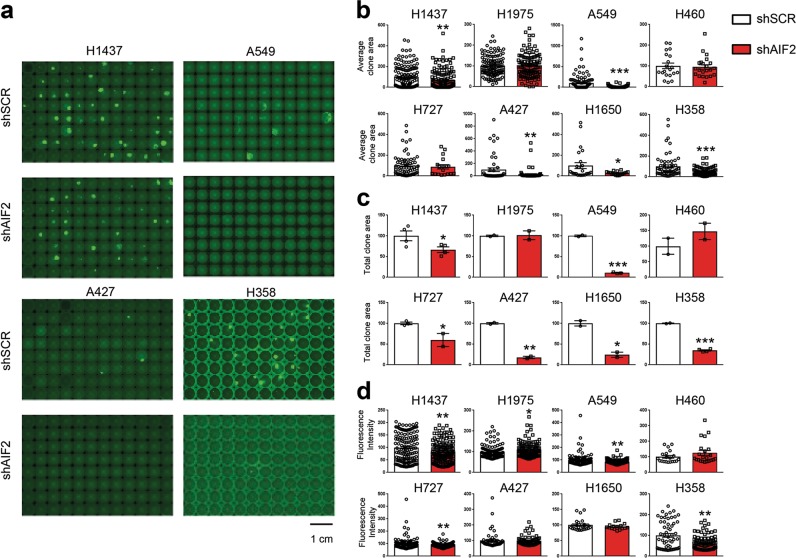
Stable *Aif* depletion impairs colony formation of both *KRAS* WT and *KRAS*-mutated human lung cancer cells. *KRAS* WT cells (H1437 and H1975) and *KRAS*-mutated human lung cancer cell (H460, H727, A427, H1650, and H358) were transduced with lentiviral vectors expressing shAIF-GFP (or shSCR-GFP as a control), and GFP-positive clones were selected by cytofluorometric sorting. A549 cells were used as an internal control. **a** Representative images of different 96-well plates seeded with one GFP-positive cell per well generated from different cell lines. The images were acquired after 7–12 day-long cell culture. **b**–**d** Quantification of average GFP^+^ clone area (**b**), total GFP^+^ clone area (**c**), average fluorescence intensity (**d**). The values obtained for the control shSCR were set as 100, and the values of shAIF were normalized to individual shSCR controls. Results are expressed as means ± SEM of a representative experiment (experiments were done in triplicates). *P < 0.05; **P < 0.01; ***P < 0.001 (Student’s *t*-test)

Stable knockdown of AIF in *KRAS*-mutant human NSCLC A549 cells compromised the expression of CHCHD4, a mitochondrial AIF interactor that is important for respiratory chain biogenesis,^8^ resulting in the reduction of proteins from the respiratory chain such as CIII-UQCRC2, CIV-COXII, and CI-NDUFB8 (Fig. 4a; Supplementary information, Fig. S4), OXPHOS, ATP generation (Fig. 4b, c), clonogenic potential, as well as proliferation (Fig. 4d, e). This effect was phenocopied by knockdown of CHCHD4 in A549 cells that also compromised the expression of respiratory chain components (but not that of AIF) and resulted in reduced proliferation and diminished clonogenic potential of these human lung cancer cells (Supplementary information, Fig. S5a–d). Transfection-enforced overexpression of a CHCHD4 variant (CHCHD4exo) that directly incorporates into mitochondria restored the clonogenic potential of AIF-depleted A549 cells (data not shown). Using the same strategy, we further analyzed whether knocking down *Aif* by means of shRNA would affect the clonogenic potential and/or proliferation of additional human lung cancer cell lines, including five different *KRAS*-mutated cell lines (H460, H727, A427, H1650, and H358), and two lung cancer lines that are WT for *KRAS* (H1437 and H1975). *Aif* knockdown significantly impaired the clonogenic capacity of H1437, H727, A427, H1650, H358, and also above-shown A549 cells (Fig. 5a–d). Moreover, individual clones originating from AIF-depleted H1437, H1975, A549, H460, and H727 cells exhibited reduced proliferation compared to their AIF-competent controls (Supplementary information, Fig. S6a–e). Importantly, none single colony could be obtained from A427, H1650, and H358 cells after AIF knockdown, indicating that AIF is essential for the proliferation of these human lung cancer cell lines. Altogether, these findings indicate that AIF-sustained OXPHOS may be essential for the optimal proliferation of both *Kras*-mutated and *Kras* WT human lung cancer cells.

### Re-expressing WT or mitochondria-anchored AIF restores lung cancer sensitivity

**Fig. 6:**
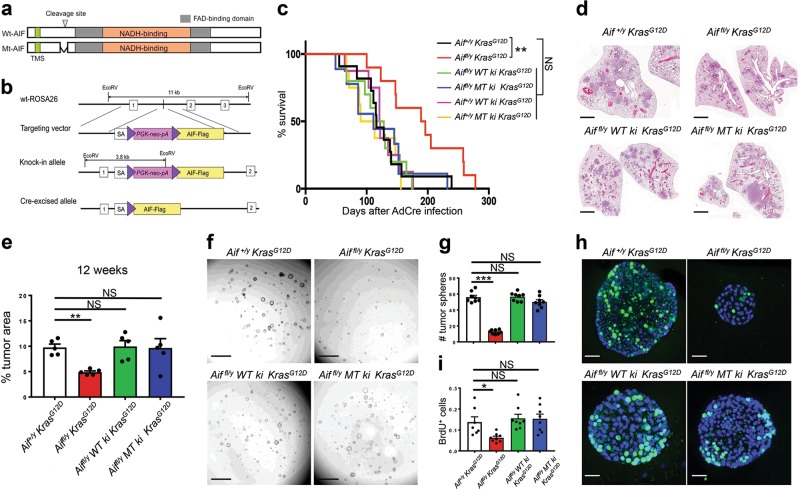
Re-expressing WT or mitochondria-anchored AIF restores lung cancer sensitivity. **a** Schematic representation of the WT AIF (WT-*Aif*) and mitochondria-anchored mutant AIFΔ96-110 (MT-*Aif*). TMS, transmembrane sequence. The cathepsin/calpain cleavage site is indicated. **b** Knock-in targeting strategy to insert WT-*Aif* and MT-*Aif* transgenes into the ROSA26 locus. Exons are shown as 1, 2, and 3. HSV-tk = herpes simplex promoter; PGK-neo-PA = neomycin cassette for selection; AIF-FLAG = AIF (WT or mutated) with a 3 × FLAG tag, *Eco*R V = restriction sites for Southern blotting. **c** Kaplan Meier survival plots for *Aif*^*+/y*^ *Kras*^*G12D*^ (*n* = 11), *Aif*^*fl/y*^ *Kras*^*G12D*^ (*n* = 10), *Aif*^*fl/y*^ *WT ki Kras*^*G12D*^ (*n* = 10), *Aif*^*fl/y*^ *MT ki Kras*^*G12D*^ (*n* = 9), *Aif* ^*+/y*^ *WT ki Kras*^*G12D*^ (*n* = 9), and *Aif*^*+/y*^ *MT ki Kras* ^*G12D*^ (*n* = 8) mice. **P *<* 0.01; NS, not significant (log rank test). **d** Representative lung sections (H&E staining) of *Aif*^*+/y*^ *Kras*^*G12D*^, *Aif*^*fl/y*^ *Kras*^*G12D*^, *Aif*^*fl/y*^ *WT ki Kras*^*G12D*^ and *Aif*^*fl/y*^ *MT ki Kras*^*G12D*^ mice, analyzed at 12 weeks after Ad5-CMV-Cre infection. Scale bar, 2 mm. **e** Quantification of overall tumor burden in the indicated cohorts analyzed 12 weeks after Ad5-CMV-Cre inhalation (*n* = 5 for each genotype). Three planes from each lung were scored automatically by an algorithm programmed and executed using the Definiens software suite program. Data are shown as means ± SEM. **P *<* 0.01; NS, not significant (two-way ANOVA analysis, Dunnett’s multiple comparisons test). **f** Representative images of tumor spheroids derived from purified *Aif*^*+/y*^ *Kras*^*G12D*^ and *Aif*^*fl/y*^ *Kras*^*G12D*^ primary lung tumor cells. Images were acquired 4 days after cells were seeded in Matrigel (5000 primary tumor cells per well). The experiment was designed with 6 replicates for each condition and repeated with 3 different mice for each group. Scale bar, 1 mm. **g** Quantitative analysis (means±SEM) of tumor spheroid numbers described in **f**. ***P *<* 0.001; NS, not significant (Unpaired, two-sided *t*-test). **h** Representative images for BrdU staining of tumor spheroids derived from *Aif*^*+/y*^ *Kras*^*G12D*^ and *Aif*^*fl/y*^ *Kras*^*G12D*^ primary lung tumor cells seen as in **f**. BrdU labeling (10 μM/mL) was performed for 2 h. Experiments were performed with 6 replicates for each condition and repeated with 3 different mice for each genotype. Sections were counter-stained with DAPI. **i** Quantifications (means ± SEM) of BrdU^+^ cells within tumor spheroids shown in **h**. *P *<* 0.05; NS, not significant (Unpaired, two-sided *t*-test)

So far, we have shown that loss of AIF markedly delays the onset and progression of oncogenic *Kras*^*G12D*^-driven lung cancer and that *Aif*-deficient pneumocytes exhibit a Warburg-like bioenergetic profile. However, AIF has a dual role, depending on its subcellular localization: (i) as a positive regulator of OXPHOS in mitochondria, and (ii) as a caspase-independent death effector when released from mitochondria, a process that requires permeabilization of the mitochondrial outer membrane as well as proteolytic cleavage of the AIF protein.^17^ To distinguish between these two roles, we constructed two different knock-in (ki) mouse lines, namely (i) WT ki mice that express a WT *Aif* transgene inserted into the ROSA26 locus (*R26*^*AIF-WT/+*^), preceded by a *Lox-Stop-Lox* cassette, and (ii) MT ki mice that express mutant *Aif* Δ96-110 inserted into the same locus (*R26*^*AIF-Mut/+*^). This design ensures that both transgenes are only introduced once into the genome, do not cause collateral mutations, and are transcribed and regulated in a similar fashion (Fig. 6a, b). AIFΔ96-110 cannot be cleaved by cathepsins/calpains and thus is retained in mitochondria, precluding its translocation to the cytosol and the nucleus.^17^ Accordingly, in cell culture experiments, WT AIF translocates to the nucleus following a death stimulus, whereas AIFΔ96-110 remains in the mitochondria and hence cannot participate in lethal signaling pathways.^17^ Transgenic WT *Aif* (*WT ki*) and *AifΔ96-110* (*MT ki*) were indistinguishable in their capacity to reverse the embryonic lethality that is observed in mice bearing the actin Cre-*Aif*^*fl/y*^ genotype.^18^ In this setting (Fig. 6a, b), the endogenous *Aif* gene is universally deleted by Cre in all cell types yet replaced by transgene-encoded AIF following the Cre-mediated removal of the *Lox-Stop-Lox* cassette in the 5′ untranslated region (5′ UTR) of the transgenes. Mice expressing transgenic WT *Aif* or *AifΔ96-110* in lieu of endogenous AIF similarly displayed normal, healthy appearance (data not shown), indicating that the knocked-in WT as well as mitochondrial-anchored mutant AIFΔ96-110 can definitely rescue the lethality of *Aif* global knockout mice, i.e., both *Aif* transgenes are functional in vivo. These data also indicate that the extramitochondrial function of AIF is dispensable for its contribution to normal development.

In the next step, we introduced the *Aif* (*WT ki*) and *AifΔ96-110* (*MT ki*) transgenes into an *Aif*^*fl/y*^ *Kras*^*G12D*^ background. In the resulting mouse lines, infection with Ad5-CMV-Cre resulted in *Kras*^*G12D*^ induction, deletion of the endogenous *Aif* gene, and the additional deletion of the *Lox-Stop-Lox* cassette in the 5′ UTR of the knock-in *Aif* transgenes, thereby replacing endogenous AIF by either WT AIF or mitochondria-anchored mutant AIF (Supplementary information, Fig. S7a). As in the initial experimental cohorts, *Aif*^*fl/y*^ *Kras*^*G12D*^ mice survived significantly longer after Ad5-CMV-Cre inhalation than AIF-expressing control littermates (Fig. 6c). Re-expression of WT AIF in *Aif*-knockout mice resulted in significantly reduced survival to a level comparable to *Aif*^*+/y*^ *Kras*^*G12D*^ and *Aif*^*+/y*^ *WT ki Kras*^*G12D*^ mice. Importantly, replacement of endogenous AIF by mitochondria-anchored mutant AIF also restored the cancer phenotype of *Aif*^*fl/y*^ *Kras*^*G12D*^ mice (Fig. 6c). Control *Aif*^*+/y*^ *WT ki Kras*^*G12D*^ and *Aif*^*+/y*^ *MT ki Kras*^*G12D*^ mice behaved as *Aif*^*+/y*^ *Kras*^*G12D*^ mice with respect to overall survival and lung carcinogenesis, indicating that the transgenes had no additive effect in mice that expressed endogenous AIF. Immunoblot analysis confirmed efficient re-expression of the WT as well as mitochondria-anchored AIF protein in purified tumor cells from both *Aif*^*fl/y*^ *WT ki Kras*^*G12D*^ and *Aif*^*fl/y*^ *MT ki Kras*^*G12D*^ mice (Supplementary information, Fig. S7b). Furthermore, immunofluorescence staining revealed that re-introduced WT AIF was able to translocate from mitochondria to nuclei upon death stimulation, while AIFΔ96-110 (MT AIF) was retained in mitochondria (Supplementary information, Fig. S7c). In line with reduced survival, quantification of overall tumor burden showed that, at all time-points analyzed, re-expression of WT or mutant AIF resulted in increased lung tumor burden that was comparable to the *Aif*^*+/y*^ *Kras*^*G12D*^ cohorts (Fig. 6d, e; Supplementary information, Fig. S7d, e).

We next staged the malignant progression of lung tumors. Four weeks after Ad5-CMV-Cre infection, only few hyperplastic regions were visible in *Aif*^*fl/y*^ *Kras*^*G12D*^ mice, whereas the *Aif*^*+/y*^ *Kras*^*G12D*^ control mice already exhibited multiple hyperplastic lesions and small adenomas (Supplementary information, Fig. S8a, b). Importantly, the *Aif*^*fl/y*^ *WT ki Kras*^*G12D*^ and *Aif*^*fl/y*^ *MT ki Kras*^*G12D*^ knock-in mice developed similar numbers of hyperplastic lesions and adenomas to the *Aif*^*+/y*^ *Kras*^*G12D*^ control mice (Supplementary information, Fig. S8a, b). At 8 weeks and 16 weeks after Ad5-CMV-Cre infection, we observed markedly less tumor burden, in particular, reduced numbers of adenocarcinomas in *Aif*^*fl/y*^ *Kras*^*G12D*^ as compared to *Aif*^*+/y*^ *Kras*^*G12D*^ mice; restoration of AIF expression with WT as well as mitochondria-anchored AIF resulted in increased adenomas and adenocarcinomas, phenocopying the control mice that express endogenous AIF (Supplementary information, Fig. S8b, c). In conclusion, re-expression of WT and mitochondria-anchored AIF similarly rescues the endogenous AIF deficiency and accelerates the onset and progression of *Kras*^*G12D*^*-*driven lung cancer.

To further investigate the functional relevance of AIF and AIF-regulated OXPHOS in tumor cell proliferation as well as tumor stem-like properties, we employed a recently developed 3D tumor spheroid culture assay^19^ with purified primary pneumocytes isolated from *Aif*^*+/y*^ *Kras*^*G12D*^, *Aif*^*fl/y*^ *Kras*^*G12D*^, *Aif*^*fl/y*^ *WT ki Kras*^*G12D*^, *Aif*^*fl/y*^ *MT ki Kras*^*G12D*^ mice 6 weeks after Ad5-mSPC-Cre infection. Following seeding the same numbers of tumor cells from each genotype, we detected a major decrease in the numbers of tumor spheroids derived from *Aif*^*fl/y*^ *Kras*^*G12D*^ mice as compared to *Aif*^*+/y*^ *Kras*^*G12D*^ mice (Fig. 6f, g). Re-expression of WT and mutant AIF could almost completely restore the stem-like property to form tumor spheroids to the level of the *Aif*^*+/y*^ *Kras*^*G12D*^ control (Fig. 6f, g). We next determined the proliferation capacity of these tumor spheroids originated from AIF-competent or -deficient tumor cells by BrdU labeling. Again, we observed significantly less BrdU-positive cells in *Aif*^*fl/y*^ *Kras*^*G12D*^ tumor spheroids when compared to AIF WT, AIF WT knock-in and AIF mutant knock-in tumor spheroids (Fig. 6h, i). Addition of the OXPHOS inhibitor oligomycin efficiently blocked tumor spheroid formation at rather low concentrations (Supplementary information, Fig. S9a). Thus, AIF and AIF-regulated OXPHOS control lung cancer stem-like cell expansion and proliferation.

### Restoration of WT or mitochondria-anchored AIF eliminates mitochondrial respiration defect

**Fig. 7:**
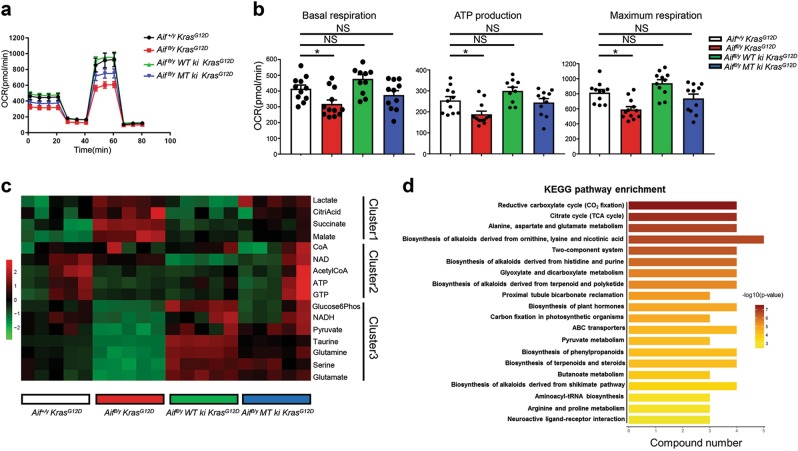
Restoration of WT or mitochondria-anchored AIF eliminates mitochondrial respiration disadvantage. **a**, **b** Representative OCR (**a**) and comparison of basal respiration, ATP production and maximal respiration (**b**) in primary purified tumor cells derived from *Aif*^*+/y*^ *Kras*^*G12D*^, *Aif*^*fl/y*^ *Kras*^*G12D*^, *Aif*^*fl/y*^ *WT ki Kras*^*G12D*^, and *Aif*^*fl/y*^ *MT ki Kras*^*G12D*^ mice 6 weeks after Ad5-mSPC-Cre inhalation. Data are shown as means±SEM. *P < 0.05; **P *<* 0.01; NS, not significant (Two-way ANOVA, Bonferroni’s post hoc test). The experiment was designed with 12 replicates for each condition and repeated with 3 different mice for each genotype. **c** Determination of metabolite content from purified tumor cells derived from *Aif*^*+/y*^ *Kras*^*G12D*^, *Aif*^*fl/y*^ *Kras*^*G12D*^, *Aif*^*fl/y*^ *WT ki Kras*^*G12D*^, and *Aif*^*fl/y*^ *MT ki Kras*^*G12D*^ mice 8 weeks after Ad5-mSPC-Cre inhalation. Data are presented in a heatmap. Experiments were performed with 3 replicates for each condition and repeated with 5 different mice for each genotype. **d** KEGG pathway enrichment assay based on metabolite concentration obtained from *Aif*^*+/y*^ *Kras*^*G12D*^, *Aif*^*fl/y*^ *Kras*^*G12D*^, *Aif*^*fl/y*^ *WT ki Kras*^*G12D*^, and *Aif*^*fl/y*^ *MT ki Kras*^*G12D*^ mice 3–4 weeks after Ad5-mSPC-Cre inhalation. Red box indicates the tricarboxylic acid (TCA) cycle, one of the top enriched pathways after AIF depletion

The re-introduction of either WT or mitochondria-anchored AIF into *Aif*^*fl/y*^ *Kras*^*G12D*^ pneumocytes could enhance their respiratory capacity to levels comparable to the *Aif*^*+/y*^ *Kras*^*G12D*^ control (Fig. 7a, b; Supplementary information, Fig. S9b). To extend this characterization, we performed comparative targeted metabolomic analysis with purified tumor cells that were freshly isolated from *Aif*^*+/y*^ *Kras*^*G12D*^, *Aif*^*fl/y*^ *Kras*^*G12D*^, *Aif*^*fl/y*^ *WT ki Kras*^*G12D*^, and *Aif*^*fl/y*^ *MT ki Kras*^*G12D*^ mice. In line with the bioenergetic profiles, AIF-deficient cells harbored significantly less pyruvate and NADH, but much more lactate (Fig. 7c), suggesting that the OXPHOS was indeed inhibited after AIF depletion. Importantly, intermediate metabolites belonging to the tricarboxylic acid cycle (TCA cycle), including citrate, malate, and succinate, were highly augmented in AIF-deficient cells (Fig. 7c), as further validated by Kyoto Encyclopedia of Genes and Genomes (KEGG) pathway enrichment analysis (Fig. 7d). All the metabolic alterations associated with *Aif* deficiency could be fully or partially restored by re-expressing WT or mitochondria-anchored AIF (Fig. 7c).

### AIF is frequently overexpressed in human lung tumors and high AIF expression is associated with poor survival

**Fig. 8:**
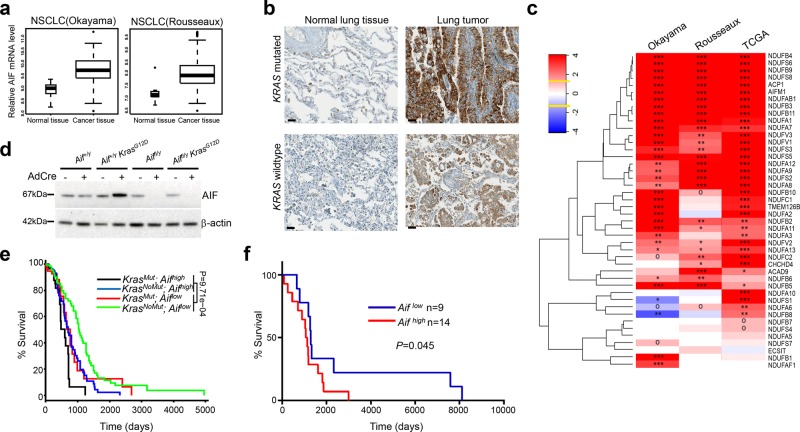
AIF is frequently overexpressed in human lung tumors, and high AIF expression is associated with poor survival. **a** Comparison of AIF mRNA expression level between normal lung tissue and lung tumor tissue derived from NSCLC patients in two independent studies, namely Okayama dataset (GEO: GSE31210) and Rousseaux dataset (GEO: GSE30219). *P*-value was obtained by Student’s *t*-test (linear model). **b** Representative AIF protein expression as determined by immunohistochemistry in lung tumors and tumor-adjacent normal lung tissue from *KRAS*-mutated and *KRAS* WT lung cancer patients. Scale bar, 20 μm. **c** Differential expression analysis of individual components from respiratory chain complex I, comparing tumor and normal lung tissues. Data were generated from the Okayama, Rousseaux and TCGA databases, respectively. Colors represent assigned log_10_ of *P*-values extracted from *t*-tests (red for overexpression, blue for underexpression in tumor compared to the adjacent normal tissue). Yellow lines in the color key (top left) represent the significance thresholds of ±log_10_ (0.05). Heatmap cells are annotated according to the statistical significance: ***P < 0.001, **P < 0.01, *P < 0.05, o*P* < 0.1. **d** Immunoblot analysis of AIF expression in primary pneumocytes purified from *Aif*^ *+* */y*^, *Aif*^*+/y*^ *Kras*^*G12D*^, *Aif*^*fl/y*^, and *Aif*^*fl/y*^ *Kras*^*G12D*^ mice which were infected with or without Ad5-mSPC-Cre (MOI = 100) for 8 days. β-actin is shown as a loading control. **e** Overall survival curves according to *KRAS* mutational status and AIF expression status in lung TCGA datasets (RNAseq). The patients were divided into four groups: *KRAS*^*Mut*^; *Aif*^*high*^ (*n* = 95); *KRAS*^*Mut*^*; Aif*^*low*^(*n* = 78); *KRAS*^*NoMut*^*; Aif*^*high*^ (*n* = 182); and *KRAS*^*NoMut*^*; Aif*^*low*^ (*n* = 282). To evaluate the overall effect of AIF, we pooled *KRAS*^*Mut*^ and *KRAS*^*NoMut*^ patients together and *P* value indicated in the panel was obtained by applying a cox model. Patients were also stratified according to the *KRAS* mutation status and evaluated by constructing a stratified cox model; *P* = 0.00673 (*KRAS*^*NoMut*^*; Aif*^*high*^ vs *KRAS*^*NoMut*^*; Aif*^*low*^) and *P* = 0.0438 (*KRAS*^*Mut*^*; Aif*^*high*^ vs *KRAS*^*Mut*^*; Aif*^*low*^). **f** Overall survival curves stratified by AIF protein expression levels, as determined by immunohistochemistry. The patient cohort was divided into two groups: *Aif*^*high*^ (*n* = 14) and *Aif*^*low*^ (*n* = 9). Due to the limited patient numbers, we pooled *Kras*^Mut^ and *Kras*^*NoMut*^ patients. *P* = 0.045 (Log rank test)

Finally, we explored the clinical relevance of AIF in human lung cancer by retrospectively analyzing published datasets. Transcriptome studies of both lung adenocarcinoma (Okayama dataset; GEO: GSE31210)^20,21^ and different lung cancer subtypes (Rosseaux dataset; GEO: GSE30219)^22^ demonstrated a significant upregulation of the *AIF* mRNA levels in lung cancer tissue compared to normal tissue (Fig. 8a). To validate this finding at the protein level, we investigated AIF expression in human lung cancer samples by immunohistochemistry. In line with the previous findings, we observed elevated AIF expression in the tumor tissue from both *Kras*-mutated and *Kras* WT lung cancer patients compared to their normal lung tissue (Fig. 8b). We did not detect significant differences in AIF expression among primary lung cancer, locally advanced lung cancer or metastatic lung cancer, which might be due to the limited number of patient samples with genetic workup as well as with long-term follow-up. Since AIF is an important component of respiration chain complex I, we then explored whether other components would show a similar upregulation in lung tumor tissue compared to normal lung tissue. We found that, in three different NSCLC cohorts (Okayama, Rosseaux and The Cancer Genome Atlas, TCGA), not only AIF but also the large majority of genes encoding the complex I subunits or their assembly factors were significantly overexpressed in NSCLC tissues compared to normal adjacent lung tissue (Fig. 8c). Accordingly, when we infected pneumocytes isolated from *Aif*^*+/y*^ and *Aif*^*+/y*^ *Kras*^*G12D*^ mice with Ad5-mSPC-Cre, a significant induction of AIF expression was detected 8 days after *Kras*^*G12D*^ activation; however, this effect was not observed in non-transformed pneumocytes expressing WT *Kras* (Fig. 8d). Furthermore, we evaluated the correlation between AIF expression and overall survival by analyzing RNAseq data from the TCGA dataset. High AIF expression was negatively correlated with poor prognosis, irrespective of the *KRAS* mutational status (Fig. 8e), suggesting that AIF overexpression is a common event in lung cancers associated with poor prognosis. In accord, patients bearing NSCLCs that express low levels of AIF protein, as determined by immunohistochemistry, exhibited a longer survival than patients bearing AIF^high^ tumors (Fig. 8f), supporting the conclusion that high AIF expression is associated with poor prognosis.

## Discussion

Among the established hallmarks of cancer are a reduced propensity of the tumor cells to die as well as an altered metabolism,^23^ and these two characteristics may be intertwined in mechanistic terms.^24^ Resistance against cell death allows tumor cells to avoid elimination subsequent to the activation of cell-intrinsic pathways or in response to a hostile microenvironment. Tumor cell metabolism is rewired to facilitate the generation of biomass and consequent proliferation. Based on these premises, we expected that elimination of AIF should have stimulated *Kras*^*G12D*^-driven lung oncogenesis because the absence of AIF would (i) render the cells resistant against (at least some) lethal stimuli and (ii) induce a Warburg-like metabolic reprogramming due to the partial inhibition of OXPHOS. In contrast to our expectations, however, AIF favored *Kras*^*G12D*^-driven carcinogenesis, and this effect can be attributed to the metabolic function of AIF, because a mitochondrion-retained, apoptosis-deficient mutant of AIF could replace WT AIF with respect to its pro-tumorigenic activity.

One hallmark of cancer is the reprogramming of energy metabolism. Already 90 years ago, Otto Warburg presented evidence that cancer cells preferably use glycolysis for ATP production and produce lactate from glucose even under normoxic conditions.^11^ Therefore, one major therapeutic strategy for cancer treatment is to inhibit glycolysis in cancer cells and to promote OXPHOS, forcing the cell into a more “normal” metabolism which would presumably hinder cancer cell survival and growth.^25^ Drugs like 2-DG and lonidamine targeting the enzyme hexokinase, as well as Cap-232/TLN-232, an agent that targets the last step of glycolysis by inhibiting pyruvate kinase, are in clinical trials for a variety of solid tumors.^26,27^ Intriguingly, in our *Kras*^*G12D*^-driven lung cancer model, the switch to glycolysis does not promote tumor growth and is disadvantageous for the cancer. These results are in accord with the fact that AIF expression is not lost in human cancer, irrespective of the *Kras* mutational status, probably reflecting the need for AIF to sustain the bioenergetics of malignant cells. Moreover, several recent studies have suggested that unlike many other cancers, lung tumors are highly oxidative and OXPHOS is indeed required for lung cancer development.^28–30^ Several specific inhibitors of OXPHOS including BAY 87-2243,^31,32^ IACS-010759^33,34^ and VLX600^35^ have been shown to mediate anticancer effects in suitable preclinical models. It is important to note that, in our study, OXPHOS was genetically modified in cancer cells only, while the aforementioned pharmacological studies cannot distinguish whether OXPHOS inhibition in neoplastic or stromal (including immune) cells accounts for the therapeutic effects of the inhibition of mitochondrial respiration. That said, cancer cell-specific AIF knockout only partially inhibited OXPHOS, meaning that it did not completely disrupt mitochondrial bioenergetics, yet yielded tangible effects on tumor incidence and progression.

Based on the aforementioned observations, it may be interesting to explore the possibility to inhibit AIF as well as the metabolic pathways that depend on it, in particular complex I of the respiratory chain^36^ with the scope of limiting malignant growth.

## Materials and Methods

### Mice

*Aif*^*floxed*^ mice were generated by homologous recombination.^3^ Exon 7 of the *Aif* gene was targeted. These mice were backcrossed for at least 10 generations onto a C57/Bl6 background and then crossed to *LSL-Kras*^*G12D*^^12^ mice to generate *Aif*^*fl/y*^ *LSL-Kras*^*G12D*^ and *Aif*^*+/y*^ *LSL-Kras*^*G12D*^ littermate mice. WT AIF knock-in mice (AIF-3× FLAG; *WT ki*) and mitochondria-anchored AIF knock-in mice (AIFΔ96-110/3× FLAG; *MT ki*) were generated using Rosa26 locus gene targeting.^37^ These lines were backcrossed 10 times onto the C57/Bl6 background and further crossed to *Aif*^*fl/y*^ *LSL-Kras*^*G12D*^ and *Aif*^*+/y*^ *LSL-Kras*^*G12D*^ mice (see breeding scheme in Supplementary information, Fig. S7a). Genotypes were determined by PCR. Only littermate mice were used in all experiments. All our mice were maintained according to ethical animal licenses complying with Austrian and European legislation.

### Induction of lung cancer

Inhalation of 6–8-week-old mice with Ad5-CMV-Cre (VVC-U of Iowa-5) or Ad5-mSPC-Cre (VVC-Berns-1168) viruses was performed as previously reported.^38^ All experimental animals were anaesthetized with 10% Ketasol/Xylasol and placed on a heated pad. An AdCre-CaCl_2_ precipitate was produced by mixing 60 μL MEM, 2.5 μL Adeno-Cre (10^10^ p.f.u./mL; University of Iowa, Gene Transfer Vector Core Iowa, USA) and 0.6 μL CaCl_2_ (1 M) for each mouse and incubated for 20 min at room temperature.

### Histology and immunohistochemistry

All lung tumors were analyzed histologically as previously reported.^38^ Briefly, lungs were cut into 2 μm sections from at least 3 different planes and stained with haematoxylin and eosin. Lung sections were scanned using a Mirax slide scanner and lung/tumor areas were scored by an algorithm programmed and executed using the Definiens software suite and visually controlled in a blinded way. For Ki67, cleaved caspase 3, AIF and SP-C immunoperoxidase staining, paraffin-embedded sections were dehydrated and antigenic epitopes were exposed using a 10-mM citrate buffer and microwaving. Sections were incubated with rabbit polyclonal anti-Ki67 (Novocastra), anti-active caspase 3 (Cell Signaling Technology, #9661), anti-AIF (Abcam, ab32516), anti-PML (provided by G. Ferbeyre) and anti-SP-C (M20, Santa Cruz Biotechnology) antibodies. Primary Ab staining was detected by peroxidase-conjugated anti-rabbit IgG (DAKO, P0448, 1:500). Positive cells were counted on 15 randomly chosen tumor areas at 100× magnifications in a double-blinded fashion. Quantitative analysis was performed using HistoQuest^Tm^ software (TissueGnostics GmbH, Vienna, Austria, www.tissuegnostics.com).

### microCT scanning

Formaldehyde-fixed lungs were stained in a solution of 1% elemental iodine and 2% potassium iodide in distilled water for 3 days. After staining, lung samples were rinsed and mounted in plastic tubes for microcomputed tomography (CT) scanning. Lung samples were scanned using a SCANCO μCT 35 (SCANCO Medical AG, Brüttisellen, Switzerland) with a source energy of 70 keV and an intensity of 114 μA using a 0.2-mm copper filter. Images were recorded with an angular increment of 0.36°. Reconstructed microCT slices measured 1,024 × 1,024 pixels (voxel size = 20 µm). Images were imported into Amira 5.3 (Visualization Sciences Group, Mérignac Cedex, France) and filtered with a three-dimensional median filter (3 × 3 × 3 kernel). To discriminate background from lung tissue, an X-ray attenuation value of *μ* *=* 0.3987 and for lung tissue from tumor tissue an attenuation value of *μ* *=* 1.2776 were used. On the basis of segmentation, lung tissue and tumor volumes were calculated.^39^

### Mitochondrial bioenergetics

Primary pneumocytes were purified and analyzed as described.^38,40^ Lungs were removed from 6–8-week-old mice, infiltrated with dispase (2 μg/mL) through the trachea, incubated for 30 min at room temperature and then manually minced and sequentially passed through 70 μm and 35 μm filters to obtain single-cell suspensions. Next, the cells were washed in minimum essential medium (α-MEM) and red blood cells were lysed using Red Blood Cell Lysis Buffer. Macrophages were removed by plating the cells on mouse IgG (0.5 mg/mL)-coated Petri dishes at 37 °C for 30 min to 1 h. Non-adherent cells were pelleted, resuspended in MEM supplemented with 10% FCS and plated again on 10 cm cell culture dishes for the removal of fibroblasts. Non-adherent pneumocytes were then plated on collagen-coated 6 cm dishes (160 μg per 6 cm; Collagen Solution (Type I, Sigma) for 2 h at room temperature in Ham’s F-12 media supplemented with 15 mM HEPES, 0.8 mM CaCl_2_, 0.25% BSA, ITS (Sigma) and 2% BSA). Following purification, 2 × 10^5^ cells were seeded on XF24 cell culture plates coated with collagen type I. Six replicates per cell type were assayed in each experiment using Seahorse technology (Seahorse Bioscience). Cells were incubated at 37 °C in a 5% CO_2_ incubator for 24 h until they were fully attached to the plate. On the day of the experiment, cells were washed and incubated in 675 μL XF Assay media (8.3 g/L DMEM base, 3.7 g/L NaCl, phenol red, 25 mM glucose, 2 mM l-glutamine, pH 7.4) for 1 h at 37 °C. Oxygen consumption was measured under basal conditions in the presence of the mitochondrial inhibitors oligomycin (0.5 μmol/L, Calbiochem) or rotenone (0.25 μmol/L, Sigma), or in the presence of the mitochondrial uncoupler FCCP (0.3 μmol/L, Sigma). Experiments were performed at 37 °C. OCR and ECAR were calculated by the oligomycin- or FCCP-induced changes in comparison to basal rates. The total protein of each well was determined by Bradford assay and used as the reference to normalize the OCR and ECAR.

### Human lung cancer cell culture, clonogenic, and proliferation assay

NSCLC A549 and A427 cells were cultured with F-12 medium; H1437, H1975, H460, H727, H1650, and H358 cells were maintained in RPM1640 medium. The medium was supplemented with 10% fetal bovine serum (FBS) and 10 mM HEPES buffer. The human lung tumor cells were infected with scrambled (SCR) shRNAs and different shRNAs targeting human AIF or CHCHD4 using the pGFP-C-shLenti vector (Origene). In case of AIF, the following two shRNA sequences were used:

(1) 5′-GCTGGAGCAGAGGTGAAGAGTAGAACAAC-3′,

(2) 5′-CAGCCACCTTCTTTCTATGTCTCTGCTCA-3′.

For CHCHD4 downregulation, the following three shRNAs were used:

(1) 5′-GGTACTACCACAGAGCTGGAGCTGAGGAA-3′,

(2) 5′-CCATTGAGGCCACTGCAACCAAAGAAGAG-3′,

(3) 5′-GAAGGATCGAATCATATTTGTAACCAAAG-3′.

Briefly, 5 × 10^4^ cells/well were seeded 24 h prior to infection with the different lentiviral particles employing a multiplicity of infection (MOI) of 5–25, following the manufacturer’s recommendations. To select infected cells, single cells were sorted into 96-well plates using FACS DIVA (Becton Dickinson, Franklin Lakes, NJ, USA). For the assessment of clonogenic capacity, a single GFP^+^ cell was seeded in each well of 96-well plates. Images of GFP^+^ live cells were acquired with a Molecular Device ImageXpress Microscope 7–12 days after sorting. Quantifications of the numbers of clones and GFP^+^ areas of clones were done with at least 96 sorted cells. To evaluate the cell proliferation, we selected stably infected clones by adding 1.5 μg/mL puromycin to the cell cultures. Stable clones were obtained by single cell sorting as above mentioned for GFP^+^ live cells selection. Cells stably expressing control shRNAs (SCR), AIF shRNAs (shAIF) and CHCHD4 shRNAs (shCHCHD4) were seeded in 96-well imaging plates (Greiner Bio-One, Kremsmünster, Austria) at densities of 500, 1000 and 2000 cells/well. Images of GFP^+^ live cells were acquired with a Molecular Device ImageXpress Microscope at the 0, 24, 48, and 72 h timepoints. Nuclei were counterstained using Hoechst 33342 (1 µM).

Clones used for proliferation assay were transfected with the plasmids pBUDneo (pBUD) or pBUDneo-MLS-CHCHD4 (pBUD-CHCHD4)^8^ by means of Lipofectamine 2000™ transfection reagent (Invitrogen, Carlsbad, CA, USA). Selection of transfected cells was achieved by adding 1 mg/mL geneticin and the selected cells were further maintained in 750 μg/mL geneticine. All cell cultures were kept at 37 °C in a humidified incubator under 5% CO_2_ atmosphere. Unless stated otherwise, media and supplements for cell culture were purchased from Thermo Fisher Scientific (Carlsbad, CA, USA).

### Determination of pH, lactate, and ATP levels

The cell culture medium was collected on days 0–3 and the pH was measured using a pH meter (Hana Instruments). For the measurements of lactate levels, the culture medium was collected and adherent cells were trypsinized and counted using a counting chamber. Lactate amount in media was determined by measuring oxidized 2,6-dichlorophenol-indophenol (DCPIP), which is reduced by phenazine methosulfate (PMS); in turn PMS is reduced by NADH produced by lactate dehydrogenase (LDH) that oxidizes lactate to pyruvate at the same time. DCPIP oxidation was spectrophotometrically measured at 600 nm. Intracellular ATP levels were measured with the Bioluminescence Assay Kit HSII (Roche). The intensity of bioluminescence can be correlated with the amount of ATP in a sample. Pneumocytes were trypsinized after 36-h incubation in culture media with glucose (5 g/L), without glucose or with glucose plus 2-DG (concentration of 0–6 mM). According to the manufacturer’s protocol, the cells were washed once in PBS, resuspended in dilution buffer and incubated for 5 min with the provided lysis buffer. Luciferin was added in the luminometre and subsequently measured by bioluminescence. A standard curve was established using several dilutions of the ATP standard provided with the kit.

### Cytofluorometry

To assess apoptosis and cell viability, pneumocytes were trypsinized and stained with PI (5 μg/mL) or 4′,6-diamino-2-phenylindole-dihydrochloride (DAPI, 5 μg/mL) and incubated at 37 °C for 20 min, followed by cytofluorimetric analysis.

### Western blotting

Western blotting was performed following standard protocols. The following primary antibodies reactive to mouse AIF (Abcam, ab32156, 1:1000), Complex I NDUFB6 (Abcam, ab110244, 1:1000), Complex I NDUFS3 (Abcam, ab110246, 1:1000), Complex I NDUFA9 (Abcam, ab14713, 1:1000), and β-actin (Sigma, F3022, 1:10,000) were used. For western blotting of human lung cancer cell lysates, we used primary antibodies specific for AIFM1 (human AIF, 4642 S, Cell Signaling Technology), CHCHD4 (HPA034688, Prestige Antibodies, Sigma-Aldrich), MitoProfile Total OXPHOS Human WB Antibody Cocktail (ab110411, Abcam) containing 5 mAbs against Complex I subunit NDUFB8 (ab110242), NDUFS7 (ab105025), NDUFA12 (ab192617), Complex II subunit SDHB (ab14714), Complex III subunit UQCRC2 (ab14745), Complex IV subunit II (ab110258), and ATP synthase subunit α as a marker for complex V (ab14748), and β-actin-HRP (mouse monoclonal, AC-15, ab49900, Abcam) as a loading control. Membranes were incubated overnight with the primary antibodies at 4 °C (diluted in 2.5% BSA in 1× TBST buffer), washed three times in TBST for 15 min each and probed with horseradish peroxidase-conjugated secondary Abs (1:5000, Promega) at room temperature for 1 h. Ab binding was visualized by enhanced chemiluminescence (GE Healthcare, RPN2106). When needed, membranes were incubated in stripping solution Restore™ PLUS Western Blot Stripping Buffer for 10 min and re-blotted according to the manufacturer’s instructions. Densitometry was conducted employing ImageJ and using β-actin to normalize for protein expression.

### Determination of metabolites

Cell pellets (10^6^ cells) were extracted using a MeOH:ACN:H2O (2:2:1, v/v) solvent mixture. A volume of 1 mL of cold solvent was added to each pellet, vortexed for 30 s, and incubated in liquid nitrogen for 1 min. The samples were then allowed to thaw at room temperature and sonicated for 10 min. This cycle of cell lysis in liquid nitrogen combined with sonication was repeated three times. To precipitate proteins, the samples were incubated for 1 h at −20 °C, followed by centrifugation at 13,000 rpm for 15 min at 4 °C. The resulting supernatant was removed and evaporated to dryness in a vacuum concentrator. The dry extracts were then reconstituted in 100 μL of ACN:H2O (1:1, v/v), sonicated for 10 min, and centrifuged at 13,000 rpm for 15 min at 4 °C to remove insoluble debris. The supernatants were transferred to Eppendorf tubes, shock frozen and stored at −80 °C prior to LC/MS analysis. One microliter of the metabolite extract was injected on a ZIC-pHILIC HPLC column operated at a flow rate of 100 μL/min, directly coupled to a TSQ Quantiva mass spectrometer (Thermo Fisher Scientific). The following transitions were used for quantitation in the negative ion mode: AMP 346 *m/z* to 79 *m/z*, ADP 426 *m/z* to 134 *m/z*, ATP 506 *m/z* to 159 *m/z*, IMP 347 *m/z* to 79 *m/z*, GMP 362 *m/z* to 211 *m/z*, GDP 442 *m/z* to 344 *m/z*, GTP 522 *m/z* to 424 *m/z*, taurine 124 *m/z* to 80 *m/z*, malate 133 *m/z* to 115 *m/z*, citrate 191 *m/z* to 111 *m/z*, pyruvate 87 *m/z* to 43 *m/z*, lactate 89 *m/z* to 43 *m/z*, NADH 664 *m/z* to 408 *m/z*, NAD 662 *m/z* to 540 *m/z*, hexose phosphates 259 *m/z* to 97 *m/z*, Acetyl CoA 808 *m/z* to 408 *m/z*, CoA 766 *m/z* to 408 *m/z*, succinate 117 *m/z* to 73 *m/z*. Glutamine 147 *m/z* to 130 *m/z*, glutamate 148 *m/z* to 84 *m/z*, serine 106 *m/z* to 60 *m/z* were measured in the positive ion mode. For all transitions, the optimal collision energy was defined by analyzing pure metabolite standards. Chromatograms were manually interpreted using trace finder (Thermo Fisher Scientific), validating experimental retention times with the respective quality controls. All measurements were within the linear range of detection.

For the metabolomics analysis, the metabolite concentration was normalized using a *Z*-score normalization method with the formula of *y* = (*x*−*α*)/*λ*, in which *x* refers to the real concentration, *α* indicates the mean value of all samples, and *λ* is the variance of all samples. The normalized concentrations of metabolites were applied to generate a heatmap, which showed the concentration difference of all metabolites. For KEGG (http://www.kegg.jp, Tokyo, Japan) pathway analysis, the clusterProfiler R package was employed.

### 3D tumor spheroid cultures

A flat round drop of Matrigel (Corning) was seeded in cell culture plates followed by incubation at 37 °C for 5 min. Primary lung tumor cells were mixed with the Matrigel and kept on ice until they were seeded onto the droplet of Matrigel in the plate in “a droplet on a droplet” fashion. The Matrigel plug was incubated at 37 °C for 30 min and then covered with cell culture medium. Images were acquired and analyzed 7 days later. For BrdU staining, tumor spheroids were incubated with 10 μM/mL BrdU at 37 °C for 2 h, followed by 3.7% formaldehyde fixation at room temperature for 15 min. Spheres were then permeabilized with PBST for 20 min and treated with 1 M HCl and 2 M HCl, followed by neutralization with a phosphate/citric acid buffer. Tumor spheroids were then stained with an anti-BrdU antibody (Abcam, ab6326) at 4 °C overnight, visualized^20^ with a fluorescence secondary antibody, and counterstained with DAPI.

### Human cohort studies

The selected datasets used in this study are: Lung-Okayama (GSE31210),^20,21^ Lung-Rousseaux (GSE30219)^22^ and Lung Cancer RNA-seq from TCGA (https://cancergenome.nih.gov/). For the TCGA dataset, we extracted the *KRAS* mutation data and the clinical survival data; mRNA counts were normalized by using the “voom” algorithm of the limma package.^41^ For differential expression between lung tumor and normal lung tissues, *t*-tests were performed. For survival analysis, Cox model tests were performed. Human immunohistochemistry (IHC) analysis was conducted in lung tumors as well as the healthy tissue adjacent to the lung tumors using a monoclonal antibody against human AIF (Abcam, ab32516). IHC staining was quantified using H-scores (range 0–300), which incorporate staining intensity (range 0–3) and the percentage of positively-stained tumor cells (range 0–100%). The distribution of AIF expression was analyzed by the square root scale method. AIF^high^ and AIF^low^ samples were defined as 20 percentiles of the highest or lowest AIF expressors; survival is presented in Kaplan Meier curves (log rank test).

### Statistics

Normally distributed data was statistically analyzed using unpaired two-tailed *t*-tests for single comparisons, and two-way analysis of variance (ANOVA) for multiple comparisons. ANOVA analyses were followed by Bonferroni’s post hoc tests. Ordinary data was analyzed using the unpaired two-tailed Mann–Whitney test. Overall survival was analyzed in Kaplan–Meier curves using a log-rank test. Microscopy images were segmented and analyzed by means of the MetaXpress (Molecular Devices) software and numerical data were further processed with R software (http://www.r-project.org/). Unless otherwise specified, data are presented as means ± SEM. The statistical tests and *P* values are indicated in each figure legend. *P* ≤ 0.05 was considered to indicate statistical significance. Numbers of mice per group used in each experiment are annotated in the corresponding figure legends as n. All the scripts used for the analysis are available upon reasonable request.

## Supplementary information

Supplementary information, Figure S1

Supplementary information, Figure S2

Supplementary information, Figure S3

Supplementary information, Figure S4

Supplementary information, Figure S5

Supplementary information, Figure S6

Supplementary information, Figure S7

Supplementary information, Figure S8

Supplementary information, Figure S9
