# Supplementary material for: AIF-regulated oxidative phosphorylation supports lung cancer development
Source: Cell Res. 2019 May 27;29(7):579–91. doi: 10.1038/s41422-019-0181-4 (PMC6796841; doi:10.1038/s41422-019-0181-4)
Supplement: Supplementary file 1 — Supplementary information, Figure S1 [file 41422_2019_181_MOESM1_ESM.pdf]

Supplementary information, Figure S1

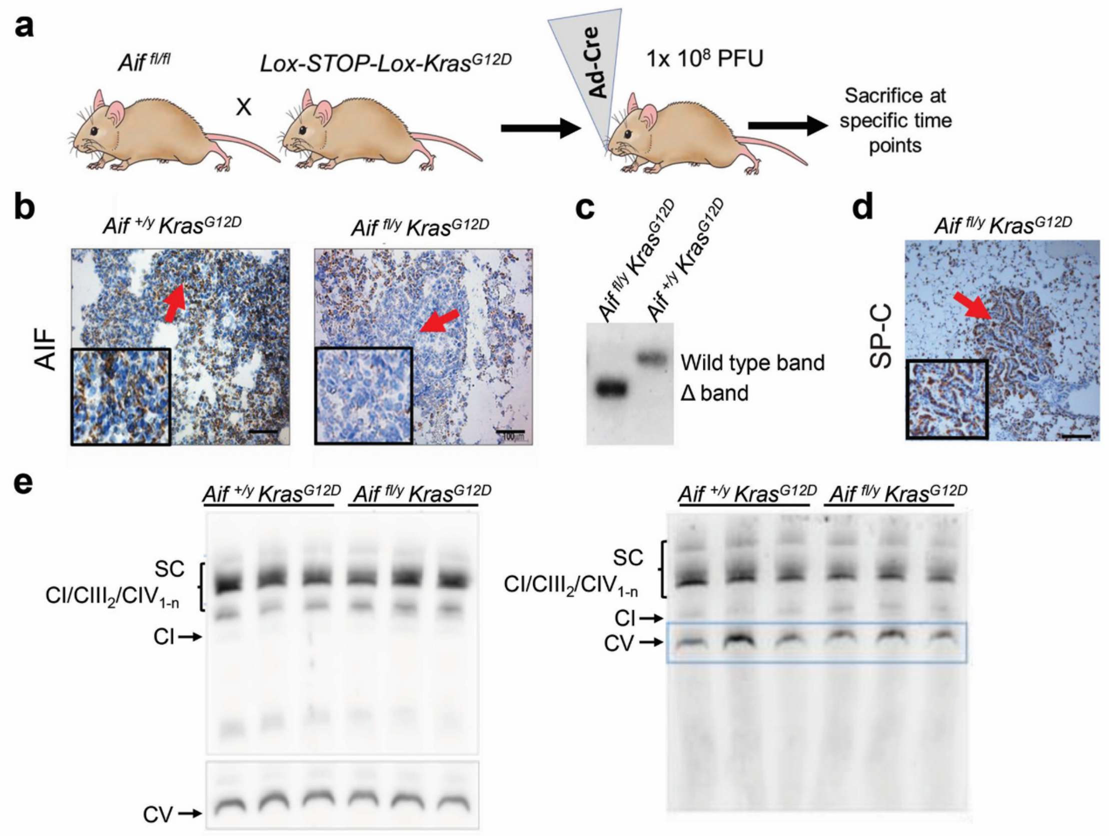

Fig. S1 *Aif* deletion in *Kras*$^{\mathrm{G12D}}$-driven lung tumors. a Breeding scheme and infection with Ad5-CMV-Cre or Ad5-mSPC-Cre. *Aif* exon7 is floxed and excision of this exons by Cre results in a frameshift of the reading frame. Ad5-CMV-Cre or Ad5-mSPC-Cre inhalation also results in simultaneous induction of oncogenic *Kras*$^{\mathrm{G12D}}$ upon removal of the stop *Lox-Stop-Lox-Kras*^{G12D} cassette. **b** Immunohistochemical staining of AIF in lung tumors from *Aif*$^{fl/y}$ *Kras*^{G12D} and *Aif*$^{+/y}$ *Kras*^{G12D} mice. Note loss of AIF protein expression in the *Aif*$^{fl/y}$ *Kras*^{G12D} tumor (arrows). Representative lung tumor sections are shown at 22 weeks after Ad5-CMV-Cre inhalation. Scale bar, 100 µm. **c** PCR analysis of laser micro-dissected lung tumor sections showing the presence of the mutant Δ band (400 bp)

in *Aif*$^{fl/y}$ *Kras*^{G12D} tumors and the wild-type band (700 bp) in *Aif*$^{+/y}$ *Kras*^{G12D} tumors. **d** Immunohistochemical staining for the alveolar type II marker SP-C (arrows) revealed that tumors arise from the infected type II pneumocytes. Data are from a representative *Aif*$^{fl/y}$ *Kras*^{G12D} adenoma 12 weeks after Ad5-CMV-Cre infection. Scale bar, 100 µm. **e** Respiratory super complex analysis of lung tumor cells isolated from *Aif*$^{+/y}$ *Kras*^{G12D} and *Aif*$^{fl/y}$ *Kras*^{G12D} mice using the BNPAGE protocol. The blot was probed with anti-NDUFA12 (left panel) and anti-NDUFS7 antibodies (right panel), respectively. Blots were also re-analyzed with anti-ATP5F1A antibody to detect CV as a loading reference. SC, super complex; CI, complex I; CV, complex V.
