# Supplementary material for: AIF-regulated oxidative phosphorylation supports lung cancer development
Source: Cell Res. 2019 May 27;29(7):579–91. doi: 10.1038/s41422-019-0181-4 (PMC6796841; doi:10.1038/s41422-019-0181-4)
Supplement: Supplementary file 2 — Supplementary information, Figure S2 [file 41422_2019_181_MOESM2_ESM.pdf]

Supplementary information, Figure S2

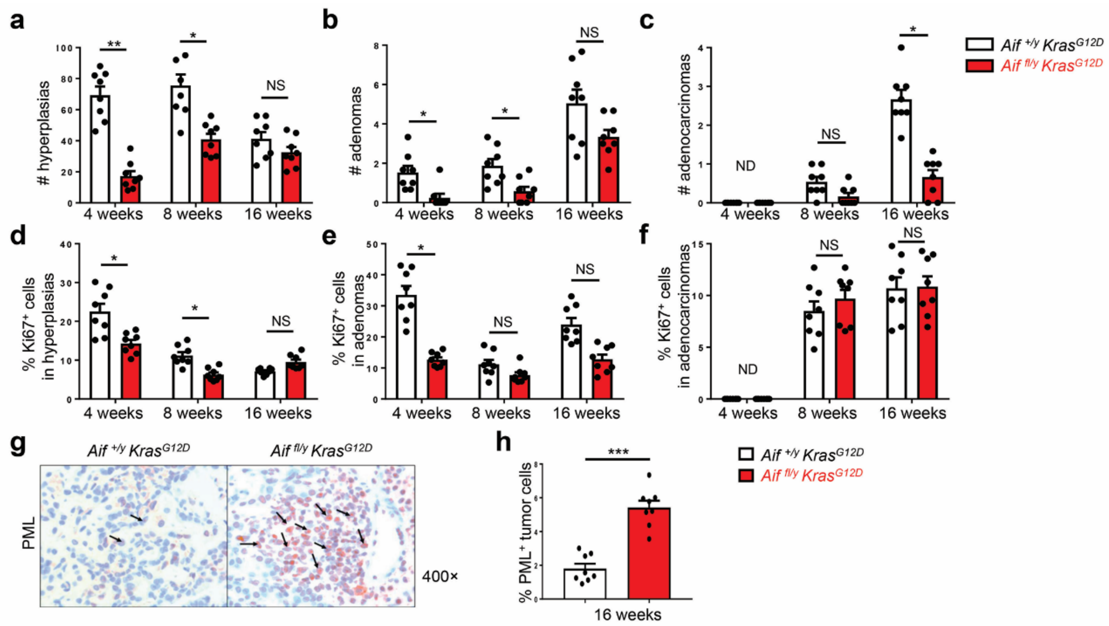

Fig. S2 Loss of AIF delays lung tumor progression. Distribution of **(a)** hyperplasias, **(b)** adenomas and **(c)** adenocarcinomas in lungs from *Aif*$^{fl/y}$ *Kras*^{G12D} and *Aif*$^{+/y}$ *Kras*^{G12D} littermates assessed 4, 8, and 16 weeks after Ad5-CMV-Cre infection. For quantification of hyperplasias, we combined hyperplastic lesions and atypical adenomatous hyperplasia (AAH). Quantification of Ki67 immunostaining in **(d)** hyperplasias, **(e)** adenomas and **(f)** adenocarcinomas in lungs from *Aif*$^{fl/y}$ *Kras*^{G12D} and *Aif*$^{+/y}$ *Kras*^{G12D} littermates assessed 4, 8, and 16 weeks after Ad5-CMV-Cre infection. **g** Representative images of PML immunostaining of tumors from *Aif*$^{fl/y}$ *Kras*^{G12D} and *Aif*$^{+/y}$ *Kras*^{G12D} littermates assessed at 16 weeks after Ad5-CMV-Cre infection. **h** Quantification of PML immunostaining in **g**. Data are shown as means ± SEM. $*P < 0.05$; $**P < 0.01$; $***P < 0.001$; NS, not significant (Unpaired two-sided *t*-test). n = 8 per genotype for each time point. Three sections per lung were analyzed. Three planes from each lung were stained with H&E and analyzed in a blinded fashion. ND, not detectable.
