# Supplementary material for: AIF-regulated oxidative phosphorylation supports lung cancer development
Source: Cell Res. 2019 May 27;29(7):579–91. doi: 10.1038/s41422-019-0181-4 (PMC6796841; doi:10.1038/s41422-019-0181-4)
Supplement: Supplementary file 3 — Supplementary information, Figure S3 [file 41422_2019_181_MOESM3_ESM.pdf]

Supplementary information, Figure S3

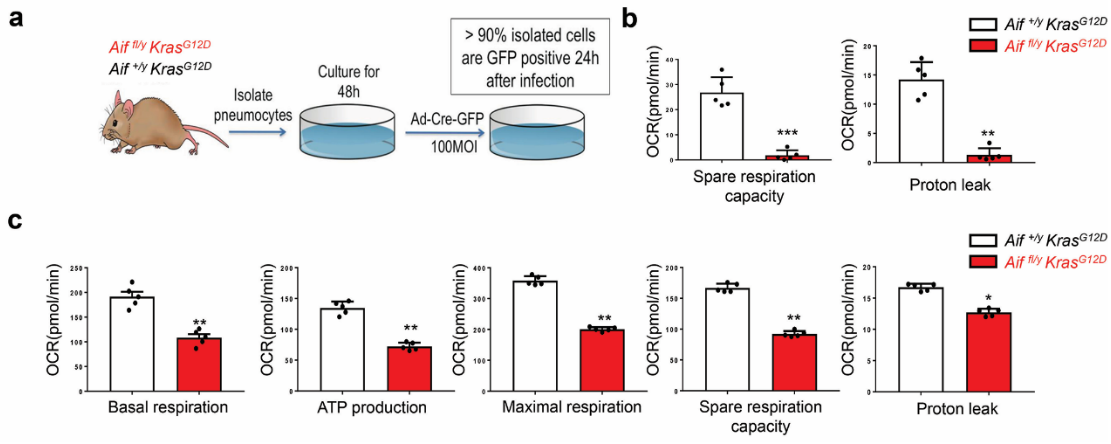

Fig. S3 Loss of AIF results in defective OxPhos. a Isolation of primary pneumocytes from $Aif^{+/y}$ $Kras^{G12D}$ and $Aif^{fl/y}$ $Kras^{G12D}$ mice. Cells were cultured for 48 h and infected with 100 MOI Ad5-CMV-Cre-eGFP; ~90% of the cultured cells were positive for GFP. **b** Comparison of spare respiration capacity and proton leakage in primary pneumocytes isolated from $Aif^{fl/y}$ $Kras^{G12D}$ and $Aif^{+/y}$ $Kras^{G12D}$ mice and consequently transfected with Ad5-CMV-Cre *in vitro.* Data using OCR Seahorse analysis are shown as means ± SEM (n = 5 per genotype). $^{**}P < 0.01$; $^{***}P < 0.001$ (Unpaired two-sided *t*-test). **c** Comparison of basal respiration, ATP production, maximal respiration, spare respiration capacity and proton leak in pneumocytes isolated 6 weeks after Ad5-CMV-Cre inhalation between $Aif^{fl/y}$ $Kras^{G12D}$ and $Aif^{+/y}$ $Kras^{G12D}$ mice. Data are shown as means ± SEM (n = 5 per genotype). $^{*}P < 0.05$; $^{**}P < 0.01$ (Unpaired two-sided *t*-test).
