# Supplementary material for: AIF-regulated oxidative phosphorylation supports lung cancer development
Source: Cell Res. 2019 May 27;29(7):579–91. doi: 10.1038/s41422-019-0181-4 (PMC6796841; doi:10.1038/s41422-019-0181-4)
Supplement: Supplementary file 4 — Supplementary information, Figure S4 [file 41422_2019_181_MOESM4_ESM.pdf]

Supplementary information, Figure S4

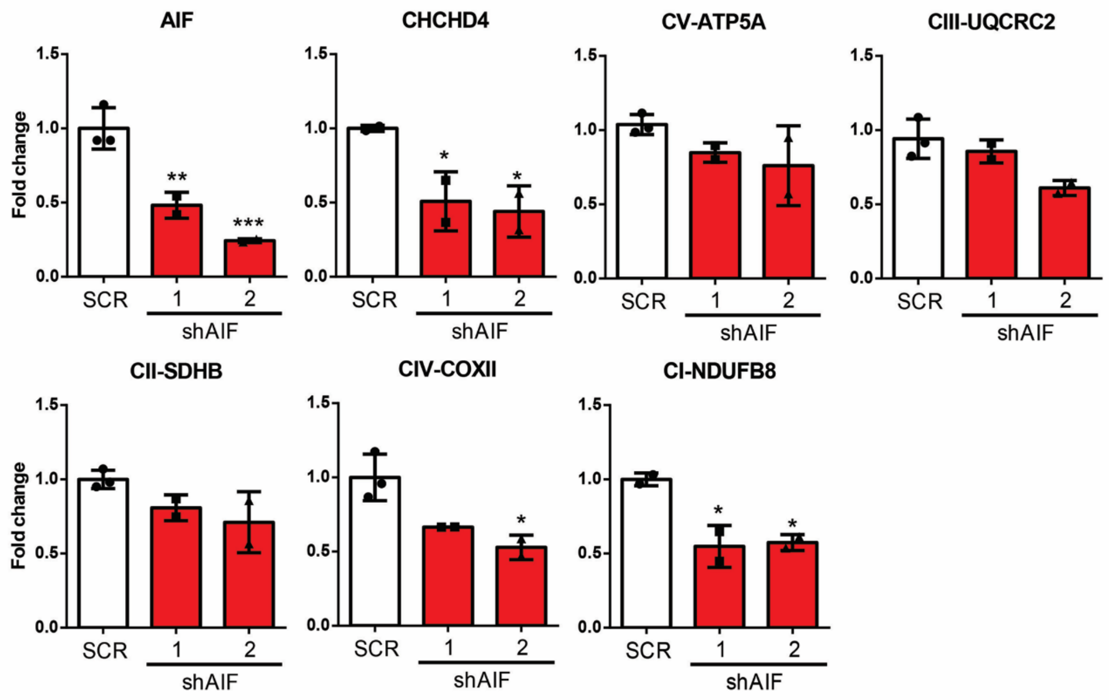

Fig. S4 Expression of respiratory chain proteins in *Aif*-knockdown human NSCLC A549 lung tumor cells. The relative protein expression levels of AIF and the respiratory chain proteins CHCHD4, CV-ATP5A, CIII-UQCRC2, CII-SDHB, CIV-COXII and CI-NDUFB8 were quantified by image analysis and normalized to the β-actin loading control after lentiviral transduction with shRNA scramble (SCR) or two different shRNA constructs targeting AIF (shAIF1 and shAIF2). Results are expressed as fold changes as compared to the scrambled (SCR) control samples. $*P < 0.05$; $**P < 0.01$; $***P < 0.001$ (Unpaired two-sided *t*-test).
