# Supplementary material for: AIF-regulated oxidative phosphorylation supports lung cancer development
Source: Cell Res. 2019 May 27;29(7):579–91. doi: 10.1038/s41422-019-0181-4 (PMC6796841; doi:10.1038/s41422-019-0181-4)
Supplement: Supplementary file 5 — Supplementary information, Figure S5 [file 41422_2019_181_MOESM5_ESM.pdf]

# Supplementary information, Figure S5

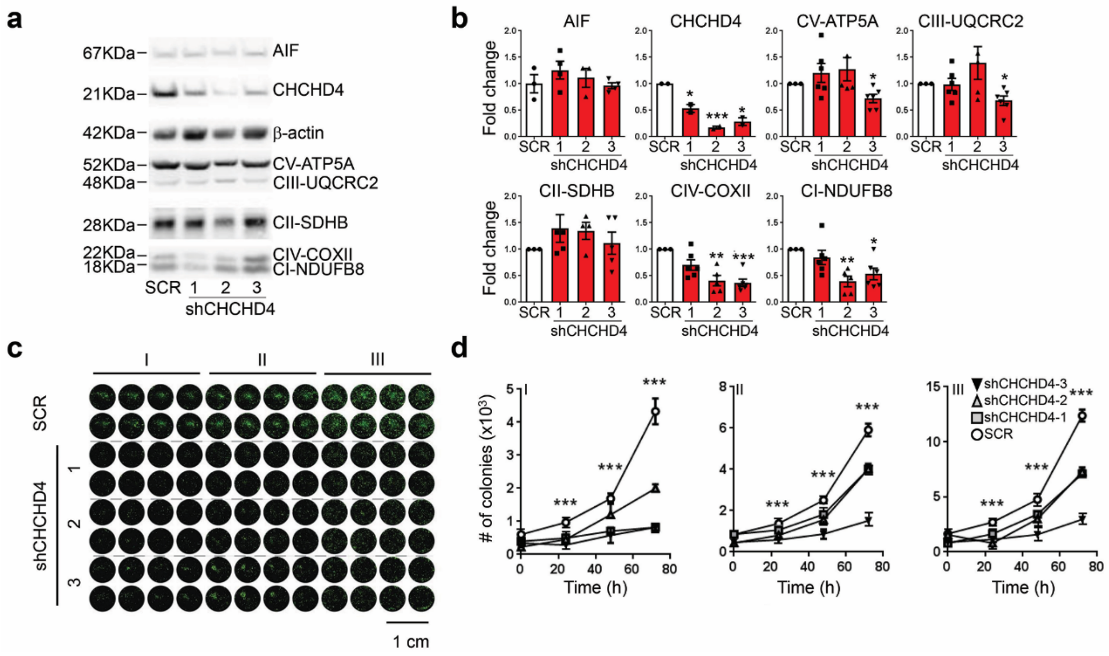


**Fig. S5 CHCHD4 depletion reduces growth of *KRAS*-mutant human A549 lung cancer cells. a** Cellular extracts from A549 clones (20 µg), generated by lentiviral transduction with shRNA scramble (SCR) and three different shRNA constructs targeting CHCHD4 (shCHCHD4-1, shCHCHD4-2 and shCHCHD4-3), were analyzed by immunoblot for the abundance of the indicated proteins. **b** Relative expression levels of the indicated proteins were quantified by image analysis and normalized to the β-actin loading control. Results are expressed as fold changes as compared to SCR control samples. **P* < 0.05; ***P* < 0.01; ****P* < 0.001 (Unpaired two-sided *t*-test). **c** Representative cell growth assay of SCR, shCHCHD4-1, shCHCHD4-2 and shCHCHD4-3 A549 lung tumor clones (I, 500 cells/well; II, 1000 cells/well and III, 2000 cells/well), analyzed by GFP fluorescence at 72 h post-seeding. **d** Quantification of the cell growth experiments. The indicated SCR, shCHCHD4-1, shCHCHD4-2 and

shCHCHD4-3 A549 clones were plated (I, 500 cells/well; II, 1000 cells/well and III, 2000 cells/well) and colony numbers quantified by GFP fluorescence at 0, 24, 48 and 72 h post-seeding. Values are means ± SEM of a representative experiment containing 16 repeats of each condition (experiment was done in triplicate with similar results). ***$P$ < 0.001 as compared to SCR cells (two-way ANOVA, Bonferroni's post hoc test).
