# Supplementary material for: AIF-regulated oxidative phosphorylation supports lung cancer development
Source: Cell Res. 2019 May 27;29(7):579–91. doi: 10.1038/s41422-019-0181-4 (PMC6796841; doi:10.1038/s41422-019-0181-4)
Supplement: Supplementary file 6 — Supplementary information, Figure S6 [file 41422_2019_181_MOESM6_ESM.pdf]

Supplementary information, Figure S6

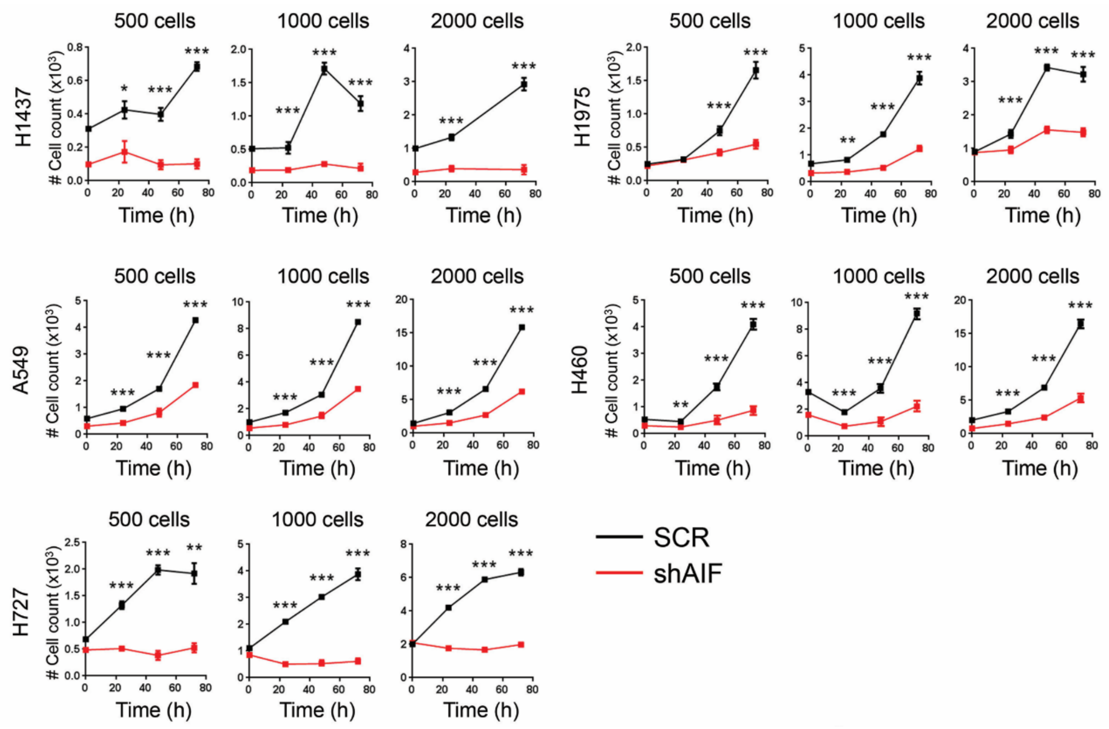

Fig. S6 ***Aif*** **knockdown inhibits human lung cancer cells proliferation.** Quantification of cell growth using 500, 1000 or 2000 cells derived from one single GFP^{+} clone harboring either shSCR or shAIF generated from the following human lung cancer cell lines: **(a)** H1437; **(b)** H1975; **(c)** A549; **(d)** H460 and **(e)** H727. Cell numbers were calculated by assessing GFP fluorescence at 0, 24, 48 and 72 h post-seeding. Values are means ± SEM of one representative experiment containing 6 repeats of each condition (experiments were done in duplicate or triplicate). *$P < 0.05$; **$P < 0.01$; ***$P < 0.001$ (Unpaired two-sided *t*-test).
