# Supplementary material for: AIF-regulated oxidative phosphorylation supports lung cancer development
Source: Cell Res. 2019 May 27;29(7):579–91. doi: 10.1038/s41422-019-0181-4 (PMC6796841; doi:10.1038/s41422-019-0181-4)
Supplement: Supplementary file 7 — Supplementary information, Figure S7 [file 41422_2019_181_MOESM7_ESM.pdf]

Supplementary information, Figure S7

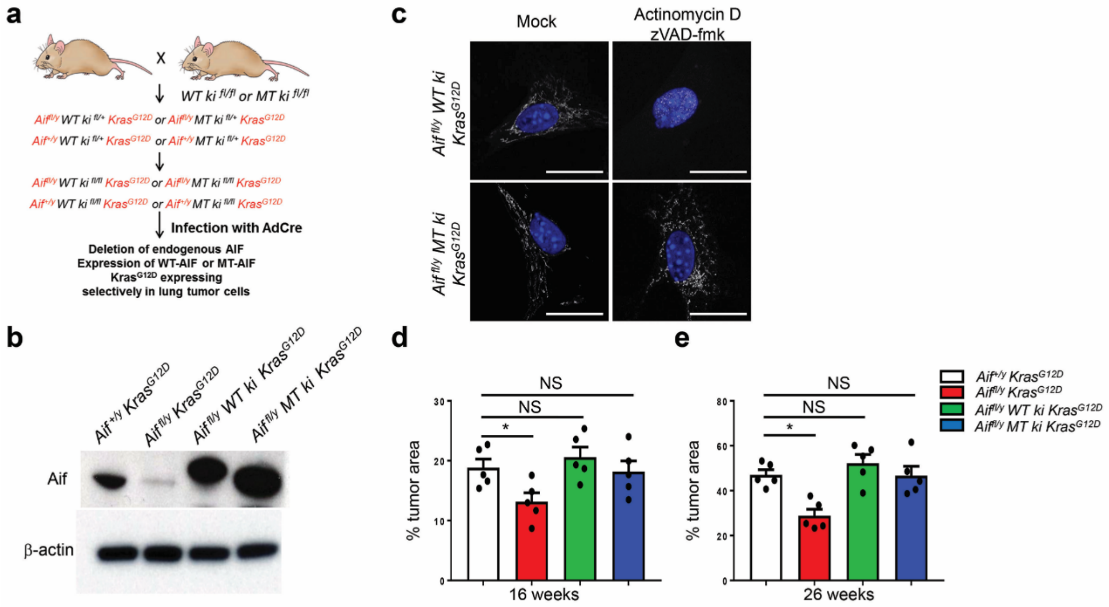

Fig. S7 Re-expression of wild-type and mitochondria-anchored AIF in *Aif*$^{fl/y}$ *Kras*^{G12D} mice. a Breeding scheme to generate mice that express *Kras*$^{\mathrm{G12D}}$, carry a tissue-specific endogenous *Aif* deletion and at the same time re-express wild-type AIF or mitochondria-anchored AIF. **b** Immunoblot analysis of AIF expression in pneumocytes purified from *Aif*$^{fl/y}$ *WT ki Kras*^{G12D} and *Aif*$^{fl/y}$ *MT ki Kras*^{G12D} mice. β-actin was used as a loading control. **c** Immunofluorescence staining of AIF expression in pneumocytes purified from *Aif*$^{fl/y}$ *WT ki Kras*^{G12D} and *Aif*$^{fl/y}$ *MT ki Kras*^{G12D} mice infected with Ad5-mSPC-Cre (MOI=100). Cells were treated with 2 µM Actinomycin D plus 10 µM zVAD-fmk for 12 h, followed by incubation with anti-Flag antibody to identify AIF location. White, Flag; blue, DAPI; Scale bar, 20 µm. **d, e** Quantification of overall tumor burden in the indicated genotypes analyzed 16 weeks **(d)** and 26 weeks **(e)** after Ad5-CMV-Cre inhalation (n = 5 for each genotype). Three planes from each lung were scored by an algorithm programmed

and executed using the Definiens software suite program. Data are shown as means ± SEM. *$P < 0.05$; NS, not significant (Two-way ANOVA analysis, Dunnett's multiple comparisons test).
