# Supplementary material for: AIF-regulated oxidative phosphorylation supports lung cancer development
Source: Cell Res. 2019 May 27;29(7):579–91. doi: 10.1038/s41422-019-0181-4 (PMC6796841; doi:10.1038/s41422-019-0181-4)
Supplement: Supplementary file 8 — Supplementary information, Figure S8 [file 41422_2019_181_MOESM8_ESM.pdf]

Supplementary information, Figure S8

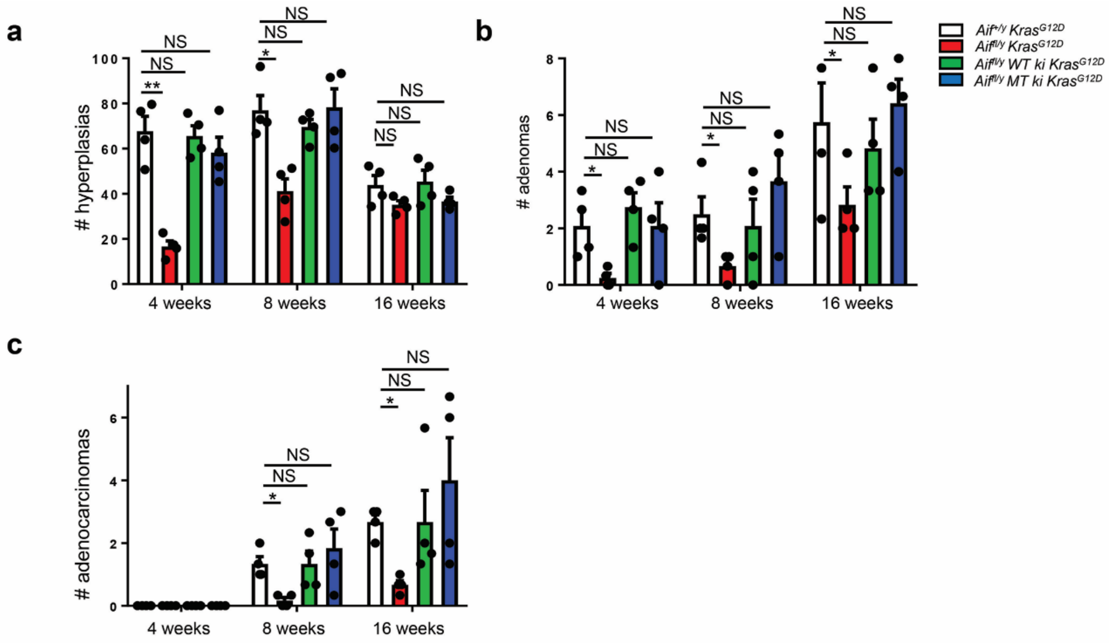

Fig. S8 Restoration of AIF expression eliminates the tumor progression disadvantage of *Aif*$^{fl/y}$ *Kras*^{G12D} mice. Percentages of **(a)** hyperplasia, **(b)** adenomas, and **(c)** adenocarcinomas in *Aif*$^{+/y}$ *Kras*^{G12D}, *Aif*$^{fl/y}$ *Kras*^{G12D}, *Aif*$^{fl/y}$ *WT ki Kras*^{G12D} and *Aif*$^{fl/y}$ *MT ki Kras*^{G12D} (n = 4 per each genotype) analyzed at 4, 8, and 16 weeks after Ad5-CMV-Cre infection. Three planes from each lung were stained with H&E and analyzed in a blinded fashion. Data are shown as means ± SEM. *$P < 0.05$, **$P < 0.01$, NS, not significant (Two-way ANOVA, Bonferroni's post hoc test).
