# Supplementary material for: AIF-regulated oxidative phosphorylation supports lung cancer development
Source: Cell Res. 2019 May 27;29(7):579–91. doi: 10.1038/s41422-019-0181-4 (PMC6796841; doi:10.1038/s41422-019-0181-4)
Supplement: Supplementary file 9 — Supplementary information, Figure S9 [file 41422_2019_181_MOESM9_ESM.pdf]

Supplementary information, Figure S9

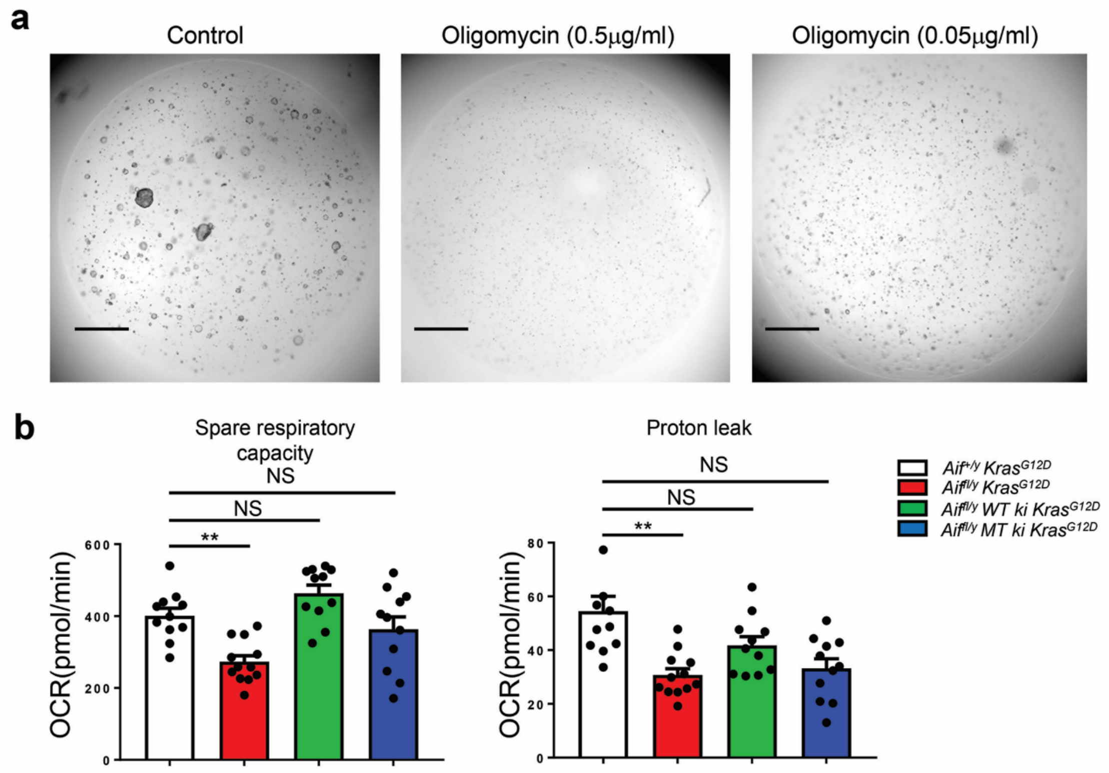

Fig. S9 Inhibition of mitochondrial respiration delays formation of lung tumor spheroids. a Representative images of tumor spheroids derived from *Aif*$^{+/y}$ *Kras*^{G12D} primary lung tumor cells, which received no treatment, or were treated with oligomycin (low dose is 0.05 μg/mL; high dose is 0.5 μg/mL). 5,000 primary tumor cells were seeded. Experiments were performed with 6 replicates for each condition and repeated with 3 different *Aif*$^{+/y}$ *Kras*^{G12D} mice. Scale bar, 1 mm. **b** Comparison of spare respiration capacity and proton leak in primary purified tumor cells derived from *Aif*$^{+/y}$ *Kras*^{G12D}, *Aif*$^{fl/y}$ *Kras*^{G12D}, *Aif*$^{fl/y}$ *WT ki Kras*^{G12D} and *Aif*$^{fl/y}$ *MT ki Kras*^{G12D} mice 3-4 weeks after Ad5-mSPC-Cre inhalation. Data are shown as means ± SEM. ***P* < 0.01; NS, not significant (Two-way ANOVA, Bonferroni's post hoc test). The experiment was designed with 12 replicates for each condition and repeated with 3 different mice for each genotype, respectively.
